# An ultra-high affinity ligand of HIV-1 TAR reveals the RNA structure recognized by P-TEFb

**DOI:** 10.1093/nar/gky1197

**Published:** 2018-11-27

**Authors:** Matthew D Shortridge, Paul T Wille, Alisha N Jones, Amy Davidson, Jasmina Bogdanovic, Eric Arts, Jonathan Karn, John A Robinson, Gabriele Varani

**Affiliations:** 1 Department of Chemistry, University of Washington, Seattle, Washington 98195-1700; 2 Department of Molecular Biology and Microbiology, Case Western Reserve University, Cleveland, Ohio 44106-4960; 3 Department of Chemistry, University of Zurich, Zurich, Switzerland CH-8057

## Abstract

The HIV-1 trans-activator protein Tat binds the trans-activation response element (TAR) to facilitate recruitment of the super elongation complex (SEC) to enhance transcription of the integrated pro-viral genome. The Tat–TAR interaction is critical for viral replication and the emergence of the virus from the latent state, therefore, inhibiting this interaction has long been pursued to discover new anti-viral or latency reversal agents. However, discovering active compounds that directly target RNA with high affinity and selectivity remains a significant challenge; limiting pre-clinical development. Here, we report the rational design of a macrocyclic peptide mimic of the arginine rich motif of Tat, which binds to TAR with low pM affinity and 100-fold selectivity against closely homologous RNAs. Despite these unprecedented binding properties, the new ligand (JB181) only moderately inhibits Tat-dependent reactivation in cells and recruitment of positive transcription elongation factor (P-TEFb) to TAR. The NMR structure of the JB181–TAR complex revealed that the ligand induces a structure in the TAR loop that closely mimics the P-TEFb/Tat1:57/AFF4/TAR complex. These results strongly suggest that high-affinity ligands which bind the UCU bulge are not likely to inhibit recruitment of the SEC and suggest that targeting of the TAR loop will be an essential feature of effective Tat inhibitors.

## INTRODUCTION

The interaction between the HIV trans-activator protein Tat and the trans-activation response element RNA ( TAR) is one of the most intensely studied protein–RNA complexes (1–6). Despite the availability of high resolution structures for TAR (2,7), along with Tat bound to the positive transcription elongation factor (P-TEFb) (8,9) and numerous biochemical studies (10), the molecular basis for Tat recognition of TAR remains largely unknown at atomic detail (11). This complex plays important roles in viral replication and emergence from latency through recruitment of the Super Elongation Complex (SEC) to the HIV promoter. Therefore, TAR has long been a target of antiviral research and a paradigmatic system to discover pharmaceuticals that target RNA (2,11–24), but no lead structure has reached the clinic due to insufficient activity in cellular models.

We previously described a class of cyclic peptide ligands for TAR, rationally designed to mimic the Arginine Rich Motif (ARM) of BIV Tat protein (14,25–28). These macrocycles were small β-hairpin mimics linked to a heterochiral D-pro-L-Pro dipeptide template to stabilize the β-hairpin fold (29,30). The most potent peptides exhibited low nanomolar binding *in vitro* (1 nM) against TAR (14), inhibited Tat-dependent transactivation against a variety of isolates that were both CCR5- and CXCR4-tropic (14,31) and were active in primary blood lymphocytes by simultaneously inhibiting Tat-dependent transcriptional elongation and initiation of reverse transcription (31).

These initial cyclic peptides discriminated effectively against structurally unrelated RNAs (e.g. transfer RNA (tRNA)), but not between closely related stem-loop structures such as the BIV TAR (28). We now report the design and discovery of a new peptide that binds to HIV TAR with low pM affinity and remarkable specificity. This peptide, JB181, was designed through careful analysis of the complex between the previous generation peptide and TAR (26), and the synthesis of a focused library that included non-canonical side chains. These characteristics are unprecedented amongst molecules that bind to RNA and suggest that this chemistry could find broader applications to other viral and eukaryotic RNA targets (32,33). However, despite readily penetrating cells and co-localizing with TAR, these peptides inhibit latent viral reactivation only weakly.

To understand the discrepancy between the pM affinity of JB181 and its micromolar cellular activity, we examined key biochemical mechanisms in latent viral reactivation along with the JB181/TAR structure. Surprisingly, we found JB181 is only weakly competitive with the core SEC (P-TEFb/Tat1:57/AFF4). In fact, JB181 binds to TAR when the SEC is also bound, displacing the Tat ARM. The Nuclear magnetic resonance (NMR) solution structure of JB181 bound to TAR provides an explanation for its binding properties and illustrates how TAR/JB181/SEC can be accommodated in a complex that displaces the Tat ARM, by inducing in the TAR loop the structure recognized by P-TEFb. These observations suggest that targeting the TAR RNA bulge will be insufficient to block SEC binding and provide key insight toward the successful design of potent inhibitors of Tat trans-activation.

## MATERIALS AND METHODS

### RNA and peptide synthesis

RNAs were transcribed *in vitro* using DNA templates from IDT (Coralville, IA), with in-house prepared T7 RNA polymerase, as previously described (34). 2AP-TAR (2-aminopurine substituted in position 24) was synthesized by IDT and purified by denaturing polyacrylamide gel electrophoresis. Peptides were prepared by Fmoc chemistry, as previously described (26). Fluorescently labeled cyclic peptides were prepared by reacting the single primary amine of lysine with 5/6-carboxyfluorescein succinimidyl ester or Alexa-647 succinmidyl ester (Thermo). Coupling reactions were completed as per manufacture protocol, purified by High Performance Liquid Chromatography (HPLC) and analyzed by Nuclear Magnetic Resonance (NMR).

### Binding assays

Electro Mobility Shift Assays (EMSA) and 2-aminopurine fluorescence (35) were used to measure peptide binding. For EMSA, all RNAs were 5′-dephosphorylated (25,26) and 5′-rephosphorylated using T4 Polynucleotide Kinase (PNK); shifts were performed and analyzed as previously described (14). The P-TEFb/Tat1:57/AFF4 binding assays were run as described (11), with protein samples generously donated by Drs Ursula Schulze-Gahmen and James Hurley, but binding buffers contained 250× fold excess yeast tRNA. JB181-bound samples were prepared with 3× excess labeled HIV-TAR prior to titration with the pre-formed P-TEFb/Tat1:57/AFF4 complex.

For the 2-aminopurine fluorescence assay, the structure of L22-TAR suggested modifying C24 would be non-perturbing. Fluorescence titration assays were performed as described (35) but the concentration of RNA was reduced to 100 fM to accommodate the very tight binding of these peptides. Fluorescence data were quantitatively analyzed with Kaleidagraph:

(1) \begin{eqnarray*}2\ \left( {\left[ R \right]{{\left[ P \right]}_t}} \right) &&= \left( {{{\left[ R \right]}_t} + {{\left[ P \right]}_t} + {K_{\rm D}}} \right)\ \nonumber\\ &&- \sqrt {{{\left( {{{\left[ R \right]}_t} + {{\left[ P \right]}_t} + {K_{\rm D}}} \right)}^2} - 4\left( {{{\left[ R \right]}_t}{{\left[ P \right]}_t}} \right)} \end{eqnarray*}

where [*R*] is concentration of RNA, [*R*]_t_ is total RNA concentration, [*P*]_t_ is total peptide concentration and *K*_D_ is the equilibrium dissociation constant.

### NMR spectroscopy, spectral assignments and structure determination

Methods for resonance assignments and structure determination of RNA:peptide complexes have been described (2,14,15). Additional details can be found in the Supplementary Data.

### Spreading infection

A 5 × 10^6^ 5.25.EGFP.Luc.M7 cells (M7-luc, gift of N. Landau(36)) grown in Roswell Park Memorial Institute medium (RPMI) containing 25 mM 4-(2-hydroxyethyl)-1-piperazineethanesulfonic acid (HEPES) (GE Life Sciences, Pittsburgh, PA), 10% Fetal Bovine Serum (FBS) (Sigma, St. Louis, MO) and 100 μg/ml normocin (Invivogen, San Diego, CA) were infected with the HIV-1 strain NL43-GFP-IRES-Nef (NLgNef) that expresses Green Fluorescent Protein (GFP) and Nef on a bicistronic Nef messenger RNA (a gift of David Levy (37)) at a low multiplicity of infection (<0.01 infectious units/cell). Twenty-four hours later, a 2-fold dilution series from 100 to 12.5 μM of JB181, cyclic L22 and linear L22 were added to 5 × 10^4^ cells/well in a 96-well plate in triplicate. Twenty-four hours post peptide addition, 50% of the cells and media were removed and replaced with media containing peptides at the same concentration that was removed. Forty-eight hours post peptide addition, the percentage of cells expressing GFP was determined by flow cytometry via BD Fortessa SORP (BD, Franklin Lakes, NJ). Flow cytometry data were analyzed using WinList 3D (Verity Software House, Topsham, ME). Graph and statistics generated using GraphPad Prism software (GraphPad, LaJolla, CA).

### Reactivation from latency

A 5 × 10^4^ cells/well of Jurkat clone E4 (in RPMI (38)) were added to a 96-well plate at the same time as a dilution series of JB181, cyclic L22 and linear L22 (0.39, 1.56, 6.25, 25, 50 and 100 μM) in triplicate. Four hours following peptide addition, tumor necrosis factor α (TNFα) (1 ng/ml, Peprotech, Rocky Hill, NJ) was added to stimulate HIV-1 transcription. Twenty-four hours later, the percentage of cells expressing GFP was determined by flow cytometry as described above.

### Peptide uptake

Frozen, de-identified human PBMCs (Allcells, Alameda, CA) were thawed and stimulated with 6 IU IL-2 (R&D Systems, Minneapolis, MN) and 10 μg/ml concanavalin A (ConA, MilliporeSigma, Billerica, MA) in RPMI. Six days post-thaw, CD4+ T cells were positively isolated using an EasySep Human CD4 positive selection kit in a RoboSep robot (#18052RF, Stemcell Technologies, Cambridge, MA). 2D10 Jurkat cells (38) were also grown in RPMI. L22 selectively labeled with 5/6-carboxyfluorescein was added at indicated concentrations at indicated time points prior to flow cytometry analysis as described above.

### Microscopy

2D10 Jurkats were treated with TNFα for 4 h prior to addition of AF647 labeled L22 for 30 min, then prepared for RNA FISH as described (39). Cells were washed three times in phosphate buffered saline (PBS) (Corning, Corning, NY) and affixed to poly-L lysine (Sigma, St. Louis, MO) coated glass coverslips for 5 min. Cells were washed once in PBS, fixed in 4% formaldehyde in PBS for 15 min, then washed three times with PBS. To permeabilize, cells were immersed in 1 ml of 70% (vol./vol.) ethanol for at least 1 h at 4°C. Coverslips were rehydrated in wash buffer (2× systemic sclerosis (SSC), 10% deionized formamide) for 5 min. Coverslips were hybridized in a humidified chamber at 37°C in hybridization buffer (10% dextran sulfate, 2 SSC and 10% deionized formamide) containing 2.5 μM RNA probes for 4–12 h. Stellaris RNA FISH probes (Biosearch Technologies, Novato, CA) to the HIV LTR as described (39). Following hybridization, cells were washed once in wash buffer at 37°C for 30 min and washed a second time in wash buffer containing 0.5 μg/ml Hoechst dye for 30 min at 37°C. Cells were washed in 2× SSC for 5 min and immediately mounted using Prolong Gold Antifade mountant (Life Technologies, Carlsbad, CA). Imaging was done on a DeltaVision RT epifluorescence microscope (Applied Precision, Inc., Issaquah, WA). Images were captured in z-series by using a charge-coupled-device digital camera, and out-of-focus light was digitally removed by deconvolution software (Applied Precision, Inc., Issaquah, WA).

## RESULTS

### Introduction of non-canonical amino acids identifies a pM macrocyclic ligand for TAR

**Figure 1.**
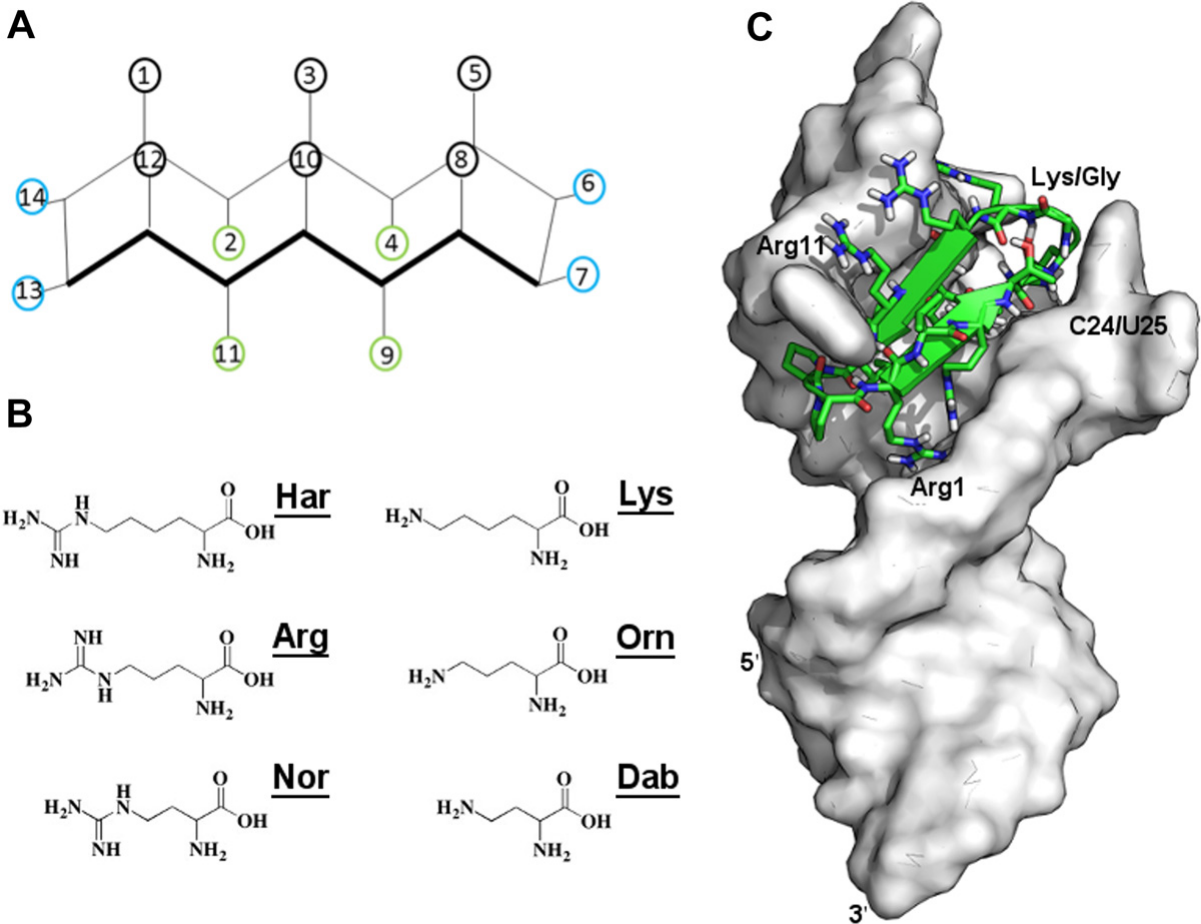
(**A**) TAR-binding cyclic peptides adopt an anti-parallel β-sheet structure with residues in black directly interacting with RNA; green residues are solvent exposed, while β-turn residues are blue, with residues 13 and 14 establishing the D-Pro/L-Pro template. (**B**) A new peptide library was constructed using extended or shortened non-standard mimics of arginine and lysine to improve contacts with RNA. (**C**) The salt bridge between Arg1 and the RNA backbone in the L22–TAR complex prevents the peptide from reaching deep into the RNA major groove.

New methods to explore macrocycle structure space and advances in delivery methods have opened therapeutic targets previously considered ‘undruggable’ (40–46). Because of their rigid scaffolds, we have pursued a class of β-hairpin macrocycle mimics (Figure 1A) that target structured RNAs and rapidly optimized them to discover low nM affinity ligands for TAR RNA (14,28,31,47,48). These cell-penetrating macrocyclic peptides discriminate well against structurally unrelated RNAs such as tRNA, but only show marginal selectivity between closely related stem-loops. Since specificity is a significant challenge in RNA-targeting therapeutics, we sought to improve the selectivity of our leads (Figure 1A) by synthesizing a relatively large positional scanning library around the L22 sequence (14), for which a structure exist, comprising natural L-amino acids, mixed chirality and non-canonical side chains (Figure 1B and Supplementary Table S1).

**Figure 2.**
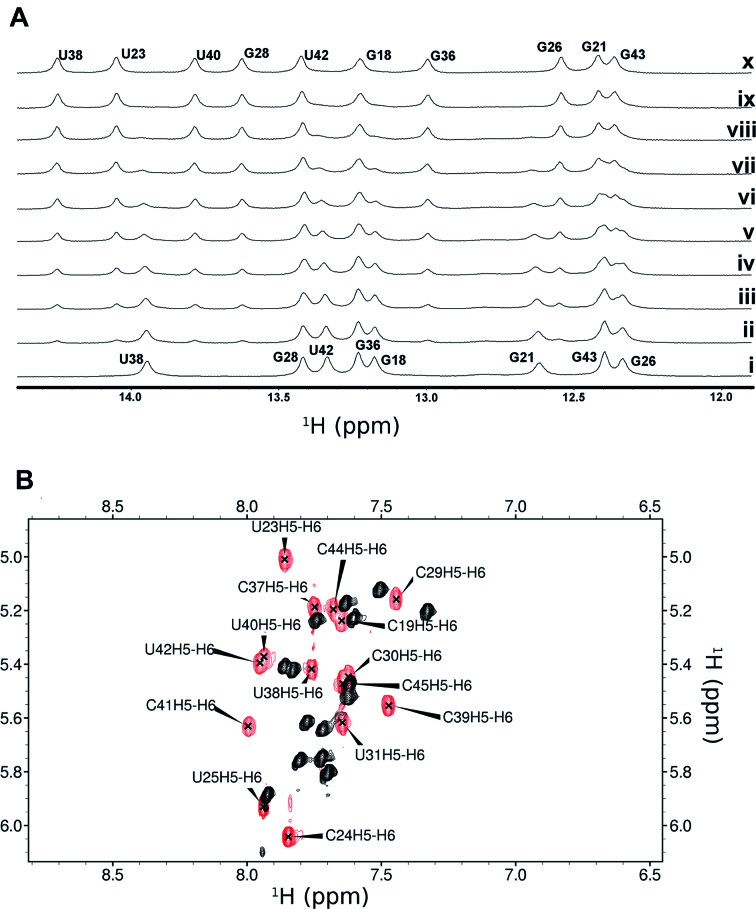
The improvement in binding affinity over other peptides of this class is highlighted by (**A**) clear slow exchange of imino resonance during a titration of HIV TAR with JB181 {1 mM RNA with 0–1.3 mM JB181 (i-x)} and (**B**) large chemical shift changes between free (black) and bound (red) TOCSY spectra. Data were recorded on a 500 MHz Bruker DRX with TCI cryoprobe, at 10°C in 95%H_2_O/5%D_2_O (A) or 25°C in 99% D_2_O (B) with the same NMR binding buffer conditions (10 mM potassium phosphate pH 6.5, 10 mM sodium chloride, 0.01mM ethylenediaminetetraacetic acid).

Introduction of L-Ornithine (Orn), or L-2,4-diaminobutyric acid (Dab) at position 1 led to the greatest improvement in binding (Table 1), followed by L-2-amino-4-guanidinobutyric acid (Nor, norarginine) at position 11. To further explore these affinity gains, we generated a very small (8 peptides) focused library holding Dab in position 1 and Nor in position 11 (Table 1). JB181 *cyclo*(Dab-Val-Arg-Thr-Arg-Lys-Gly-Arg-Arg-Ile-Nor-Ile-Dpr-Pro) emerged as the most potent sequence with sub-nM affinity, even in the presence of 250-fold excess of tRNA (*K_D_* < 200 pM by EMSA, Supplementary Figure S1). To more accurately measure the affinity of this new ligand, we substituted C24 with 2-aminopurine (2-AP) (35) and used 2-AP fluorescence to directly measure *K*_D_ = 28 ± 4 pM (Supplementary Figure S2). At last, JB181 exhibits slow exchange kinetics during NMR titrations of the imino protons (Figure 2A) and large chemical shift changes in the TOCSY (Figure 2B), indicative of ultra-high affinity.

**Table 1.** Dissociation constants for L22 derivatives binding to HIV-1 TAR RNA as determined by EMSA

Peptide	1	2	3	4	5	6	7	8	9	10	11	12	13	14	*K*_D_ (HIV), nM
**L22^14^**	R	V	R	T	R	K	G	R	R	I	R	I	p	P	30.0 ± 5.00
**JB58**	Orn	V	R	T	R	K	G	R	R	I	R	I	p	P	3.0 ± 0.3
**JB59**	Dab	V	R	T	R	K	G	R	R	I	R	I	p	P	1.0 ± 0.3
**JB181**	Dab	V	R	T	R	K	G	R	R	I	Nor	I	p	P	<0.180
**JB190**	Dab	V	R	T	R	*G*	*K*	R	R	I	Nor	I	p	P	1.6 ± 0.5
**JB186**	Dab	V	R	T	R	K	G	R	R	I	Nor	*V*	p	P	1.2 ± 0.2
**JB185**	Dab	V	R	T	R	K	G	R	R	I	R	*L*	p	P	0.5 ± 0.1
**JB184**	Dab	V	R	T	*Q*	K	G	R	R	V	Nor	V	p	P	>250

Representative binding gels for peptides can be found in SI material.

L-Ornithine (Orn), L-2,4-diaminobutyric acid (Dab) or L-2-amino-4-guanidinobutyric acid (Nor, norarginine) and D-Proline as lower-case p.

We next evaluated binding specificity by comparing two structurally and functionally related RNAs (BIV TAR and 7SK stem-loop 4). Remarkably, although these peptides were originally designed using the BIV structure as model (25,26), and the previous high affinity ligands showed little selectivity between HIV and BIV, binding of JB181 to BIV TAR occurs with *K*_D_ = 4 nM. No significant binding to 7SK-SL4 was observed at >1 μM (Supplementary Figure S1). We further evaluated JB181 binding against TAR variants identified in primary isolates, common substitutions observed over time in patients exposed to treatment (Table 2) (32). Mutating G28 or Arg5 reduces binding to JB181 nearly six orders of magnitude, and the U23C substitution or its removal abolishes JB181 binding. In contrast, variations in the apical loop sequence largely retain binding capacity to <200 pM, suggesting that any purine at these positions supports peptide binding. Mutation of A35 reduced JB181 binding but not as severely as mutations which disrupt the secondary structure, while mutating U25 to C25 showed a decrease in affinity to ∼1nM.

**Table 2.** Dissociation constants for common HIV-1 TAR mutants bound to JB181, as determined by EMSA

HIV-1 TAR	RNA Structure	*K*_D_ (HIV), nM
U23C	bulge (base triple)	no binding observed
U23_del_	bulge (base triple)	no binding observed
A35G, G28A	loop/helix junction	11 nM
G33A, G34A, G21A	loop/lower stem	70 nM
A35G, G28C	loop/helix junction	10 nM
U25C	bulge	1 nM
A35C	loop	<1.3 nM
A35_del_	loop	<1.3 nM
G32A, G36A	loop	<166 pM

### The JB181 macrocycle only weakly inhibits viral transcriptional reactivation

We previously showed that macrocyclic peptides of this class inhibit viral replication by acting both against reverse transcription and activation of proviral transcription (31). To demonstrate cell penetration and uptake, we conjugated the previous generation peptide L22 with a fluorescent dye, because the addition of Dab in JB181 adds a second primary amine making stoichiometric coupling very difficult with readily available NHS ester coupling methods, and the Dab amine is required for binding. We observed good uptake in both CD4+ T-cells and 2D10 Jurkat cells at 1 mg/ml peptide concentration in the extracellular medium (Supplementary Figure S4). High resolution images of 2D10 Jurkat cells treated with Alexa-654-L22 after induction of HIV RNA synthesis from the latent proviruses showed that cells with reduced HIV RNA and GFP protein had accumulated the highest levels of peptide, consistent with its activity as a transcriptional inhibitor (Supplementary Figure S4). Additionally, many puncta of peptide (red) overlap with cytosolic FISH-stained TAR (yellow), demonstrating that the peptides penetrate cells and co-localize with TAR within cells.

**Figure 3.**
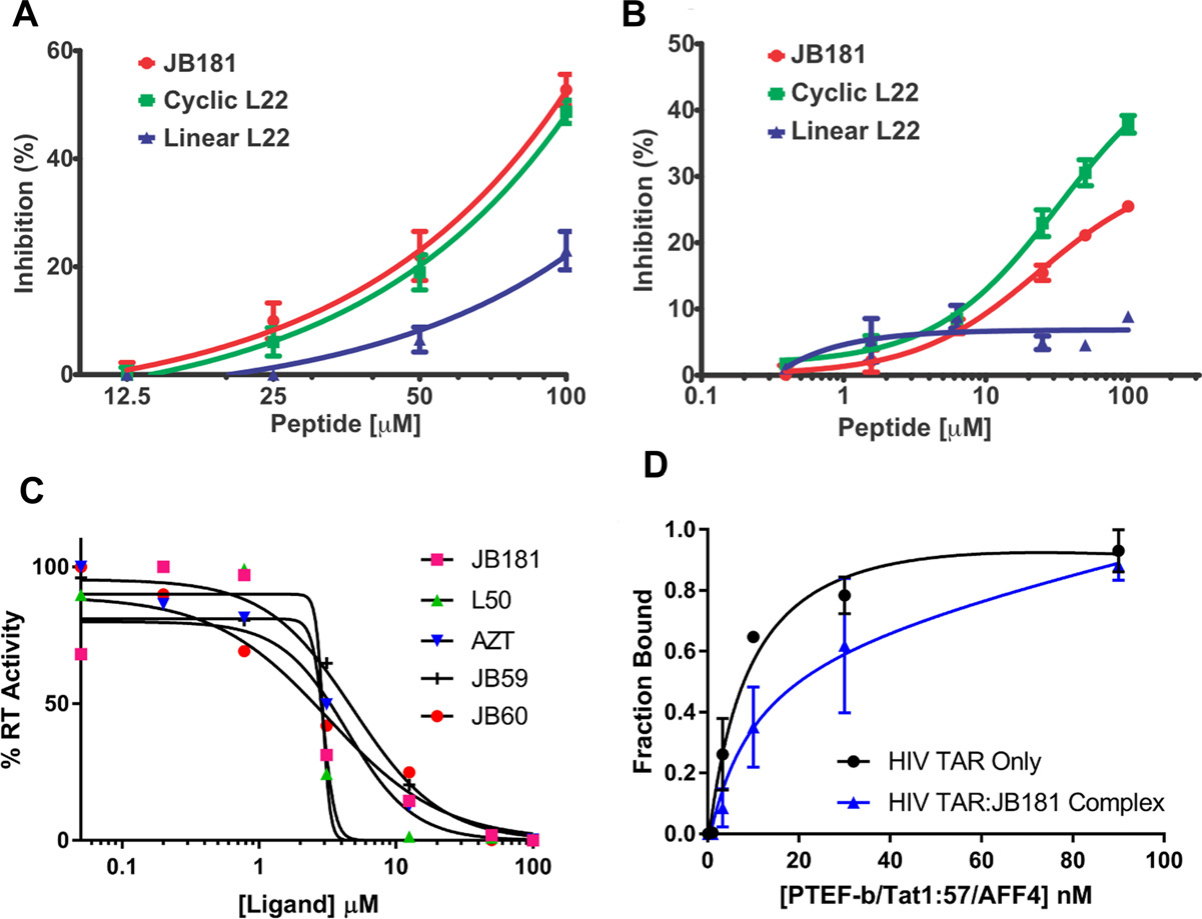
(**A**) M7-luc cells were infected with the replication competent HIV-1 clone NLgNef at very low titer. Three days post-infection, GFP expression was measured by flow cytometry. Both cyclic L22 and JB181 blocked replication and spread while linear L22, which does not bind to TAR well, showed much lower inhibition. (**B**) JB181 inhibits HIV-1 reactivation from latency. Tat-dependent expression of GFP is repressed in E4 Jurkat cells, which serve as a model of HIV latency. These cells were pre-incubated for 2 h with L22 (cyclic, green), L22 (linear, blue) or JB181 (cyclic, red) at concentrations 0.5–100 μM, then stimulated for reactivation by TNFα at 1ng/ml. Twenty-four hours later, reactivation as monitored by GFP expression was measured by flow cytometry. Both cyclic L22 and JB181 blocked reactivation while linear L22 showed no inhibition. (**C**) Biochemical RT inhibition assays were conducted as described previously (31) for peptides JB59, JB60, JB181 and L50 along with AZT. (**D**) Competition EMSA comparing affinity of the P-TEFb/Tat1:57/AFF4 complex for wild-type HIV-TAR with or without JB181 pre-bound to TAR.

To evaluate anti-viral potency, we tested compound JB181 for inhibition of viral spread (Figure 3A) by comparison with L22 and a linear peptide control (same sequence as L22 but not cyclized). The HIV-1 indicator cell line M7-luc, which expresses GFP from a Tat-dependent LTR (36), was infected with the replication-competent HIV-1 clone NLgNef, which also expresses GFP. Peptides were added 24 h post-infection and spread of infection determined after 72 h. JB181 and L22 reduced infection upward of 50% compared to no peptide controls, while the linear analog of L22 was much less active. However, we observed no significant difference between JB181 and L22, despite the >100-fold improvement in affinity and specificity.

In order to understand why large improvements in binding properties did not result in improved cellular potency, we first assessed whether our new peptides improved RT inhibition, since our previous work showed that this one mechanism of antiviral activity (31). When we compared peptides with different binding affinity for TAR, RT inhibition was approximately equal for all peptides and uncorrelated with affinity or specificity of binding (Figure 3C).

### The JB181 macrocycle only weakly inhibits SEC binding to TAR RNA

To directly study the impact of JB181 on HIV transcription, we tested our peptides in an assay in which inhibition results exclusively from blocking the interactions between TAR, Tat and the SEC, without contribution from reverse transcription (38,49). A Jurkat T cell clone (E4) containing a latent HIV provirus with a LTR-driven GFP was used to assess the impact of the peptides on latency (38,49). Following stimulation of the cells with a potent latency reversal agent (TNFα, 1 ng/ml), we found that L22 and JB181 inhibit at similar levels (Figure 3B), while the linear L22 control was much less active. Since the readout from this assay is GFP production from the provirus, these results strongly suggest that L22 and JB181 prevent Tat-mediated transcription from the LTR, but at concentrations uncorrelated with their binding affinity.

Next, we tested whether JB181 could displace the complete Tat/P-TEFb/AFF4 core SEC (11) from TAR. Surprisingly, JB181 was only weakly competitive with the P-TEFb/Tat1:57/AFF4 complex, shifting the apparent *K*_D_ by <2-fold; from *K*_D_ = 9 ±4 nM for free TAR to *K*_D_ = 17 ± 7nM for TAR bound to JB181 (Figure 3D). Analysis of the gels indicated that JB181 forms a complex with the SEC core and TAR (Supplementary Figure S6), undoubtedly by displacing the Tat ARM, since their binding sites overlap. This result provides an explanation as to why JB181 only inhibits proviral transcription at micromolar concentrations. In order to better understand why JB181 did not inhibit P-TEFb, we determined the NMR structure of JB181 bound to TAR.

### The exceptional affinity of the peptide reveals new structural features of TAR

Because of its higher affinity, NMR data quality increased significantly for JB181, with nearly 150 more NOEs observed compared to the L22 complex (14,28), whose structure was already of very high quality. Many RNA resonances which were too broad to be confidently assigned, are now clearly resolved, including a 2′-OH from A27 protected from exchange with solvent, and many previously ambiguous intermolecular NOEs (Supplementary Figures S7 and S8). A35 adopts the *syn* conformation, which was observed in half of the models for the L22 structure, and suggested as part of a sparsely populated excited state structure in free TAR (3). A new peak at 14.16 ppm corresponds to U23 in the U23-A27-U38 base triple (Figure 2). The NOE pattern demonstrates that the peptide directly interacts with U23 (Supplementary Figure S7). The base triple has long been proposed, but was not directly observed in any of the numerous peptide or small molecule-TAR complexes we exhaustively examined, including L22 (1,4,6,11,14), until a very recent crystal structure of TAR bound to a protein evolved to bind to TAR with nM affinity (50).

Each peptide backbone HN resonance is clearly resolved (Supplementary Figure S7). Four of the five guanidinium side chain resonances (all but Arg9) were confidently identified as well, indicating reduced rotation due to intermolecular hydrogen bonding. We were also able to unambiguously identify the primary amine side chains of Dab1 and Lys6, which suggest direct interactions with the RNA as well. Altogether, we were able to clearly identify at least one intermolecular NOE from each of the side chains of Dab1, Arg3, Arg5, Lys6, Arg8 and Nor11, allowing their precise placement. These observations strongly imply that the very high affinity of JB181 has pushed the TAR structure into a deep conformational free energy minimum, corresponding to its ‘bound state’, while previous Tat-derived peptides, mimics and small molecules only partially induced this conformation, which remained partly dynamic.

### Structure of the JB181 HIV-1 TAR complex

**Figure 4.**
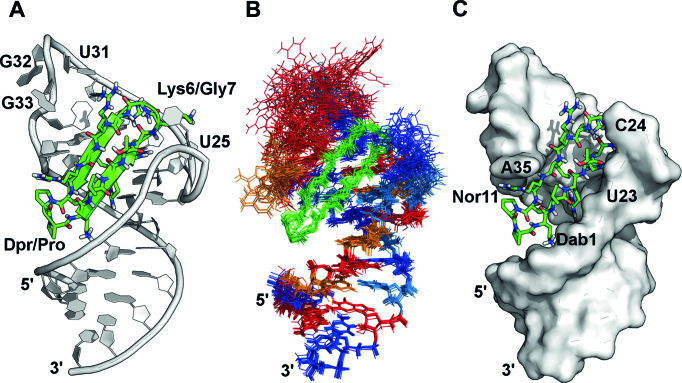
Structure of JB181 bound to HIV-1 TAR RNA, based on the experimental restraints summarized in Supplementary Table S2. (**A**) The representative (lowest energy) structure of the complex shows similar orientation to other ligands of this class. (**B**) Overlay of the 20 lowest energy structures with all atom alignment showing excellent precision even without RDC refinement (GUA = Red, ADE = Orange, CYT = Blue, URI = light blue), except for G32 and G33. (**C**) JB181 fully occupies the major groove.

The final structure was of very high-quality (all atom backbone rmsd for 10 low energy structures of 1.4 Å over the full structure) (Figure 4). Only G32 and G33 remain less precisely defined (Figure 4B) and the structure conforms well to the NMR restraints (Supplementary Table S2).

**Figure 5.**
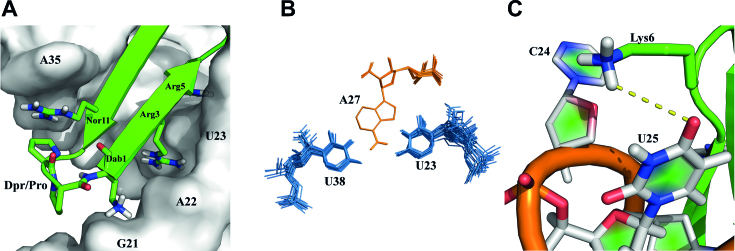
(**A**) The Dab1 salt bridge with G21 O1P shortens the distance between peptide and RNA backbones compared to Arg1 in L22 complex. This establishes a pocket between A22 and U23 for Arg3, while Arg5 sandwiches the U23 base to stabilize the base triple. (**B**) Overlay of the top 20 low energy structures shows the U23-A27-U38 base triple is rigidly defined in the JB181 complex, allowing observation of the 2′-OH from A27. (ADE = Orange, URI = light blue). (**C**) A new hydrogen bond between Lys6 and U25.

Examination of the new structure supports the conclusion that the Arg1-Dab1 substitution was most important for increasing peptide affinity. The primary amine side chain of Dab maintains a salt bridge with the RNA backbone (2.4 Å from O1P of G21 and 3.6 Å from O1P of A22). Maintaining this interaction while shortening the side chain length brings the peptide closer to the major groove by ∼2.4 Å (measured from the Cα Dab1 to P of G21) allowing the KG turn to pitch upward with respect to the helical axis (Supplementary Figure S12). This increase in pitch establishes a new binding pocket for Arg3 between A22 and U23 (Figure 4C) and sandwiches U23 between Arg5 and Arg3 (Figure 5A; a clearer close up view is provided in Supplementary Figure S13), in a manner reminiscent of the previously described arginine-fork (51). Arg3 has the sharpest Hϵ peak of all such side chain, with the NH_2_’s clearly split and well-resolved, supporting a strong interaction. The Arg3/Arg5 sandwich of U23 improves the packing of Ile10 and dramatically increases the stability of the U23-A27-U38 base triple (Figure 5B), compared to the previous L22-TAR structure (Figure S13). However, HIV-1 Tat has no equivalent hydrophobic residue within its RNA-binding domain. Thus, even in recent crystal structures, it remains unclear if a stable base triple forms in the complex between HIV Tat and TAR (11).

The upward pitch of the peptide allows formation of a new hydrogen bond with U25 (Figure 5C) supported by NOEs between Arg5 Hϵ:U25-H5 and Lys6 Hϵ:U25-H5 (Supplementary Figure S8). We recently reported this hydrogen bond in an intermediate structure of TAR bound to a linear Tat-derived peptide and to L22, linking the free and bound conformations (52). This hydrogen bond contributes to the stability the complex. As shown in Table 1, swapping the turns from KG to GK in JB190, or changing U25 to C25 (Table 2), decreased binding from ∼25 pM to ∼1.5 nM in both cases. Notably, this interaction cannot form in BIV or 7SK RNAs, as neither contains the bulged U25 residue. It is therefore likely to contribute to the specificity of JB181 toward HIV TAR as well.

## DISCUSSION

HIV-1 TAR represents a long-standing paradigm for the discovery of RNA targeting molecules (12,13,15,17,21–23,53,54) yet not one candidate has advanced even to the pre-clinical validation stage. It is often stated that a main limitation is that even high affinity ligands lack sufficient selectivity to distinguish TAR from other cellular RNAs. We report the design and synthesis of a macrocycle peptide incorporating non-canonical amino acids with unprecedented pM affinity and specificity. The remarkable binding properties of JB181 demonstrate that specific RNA recognition of relatively simple RNA secondary structures like TAR, as often found in eukaryotic non-coding RNAs, can be achieved using a structure guided approach.

**Figure 6.**
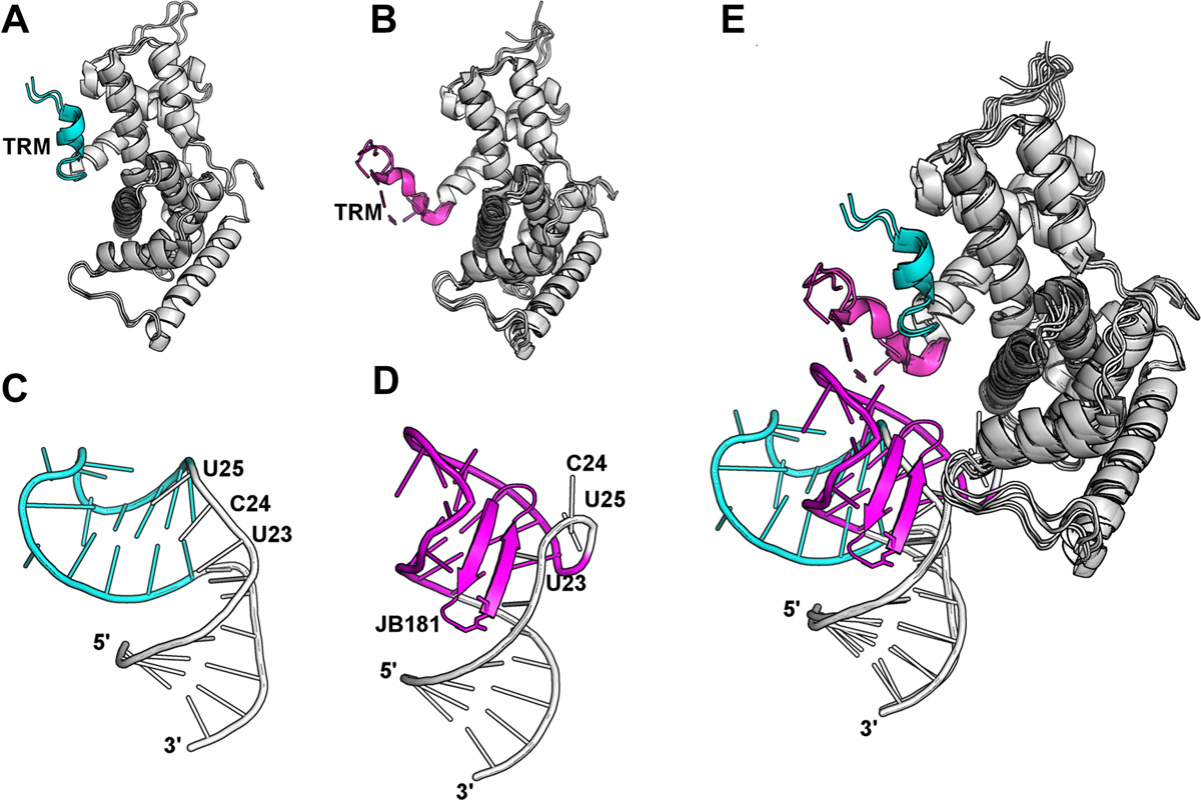
(**A** and **B**) Upon binding to CycT1 of the P-TEFb complex, Tat induces a conformational change in the C-terminal helix (residues 247–265; free = cyan, Tat-bound = magenta), which directly binds to the TAR loop and is named the TAR Recognition Motif (TRM). As recent structures show, the conformational change of the TRM is independent of TAR binding. For clarity, only the Cyt-T1 portion of each structure is shown. (Free: PDB 4EC9 and 3BLH. Bound: PDB 5L1Z, 3MI9, 3MIA and 4OR5) (**C** and **D**) At the same time, binding of peptide JB181, and presumably the Tat ARM, to the TAR UCU bulge region, reorganizes the RNA structure both globally and locally to facilitate recognition of the TAR loop by CycT1. (Free: PDB 1ANR. Bound: PDB 6D2U). (**E**) From this perspective, Tat has a dual function in inducing conformational rearrangements in both CycT1 and TAR to drive formation of a high affinity complex between TAR and the SEC.

The exceptional affinity of JB181 allows analysis of the structure of TAR to a much greater detail than possible with linear Tat-derived peptides or weaker binding macrocycles and small molecules (14,15,25–28). We directly observe the long postulated and only recently directly observed (50) U23-A27-U38 base triple, but also new features of the apical loop, namely the switching of A35 to the *syn* conformation that displays to solvent nucleotides recognized by P-TEFb and the hydrogen bonded G34:C30 base pair, both indirectly suggested by studies of NMR dynamics. Thus, TAR possesses a bi-modal structure (52), corresponding to a free and a bound conformation stabilized to different extent by different ligands, that is sparsely populated in free TAR (3,55). The JB181 peptide induces a conformation in TAR that resembles the structure of TAR bound to the Tat-ARM (Figure 6A), as we described (2), but also pre-organizes the apical loop for binding to the TAR Recognition Motif (TRM) of CycT1 (Figure 6B).

Despite the unprecedented binding characteristics, the peptide only weakly inhibits transcriptional activation and its anti-viral potency in cells does not scale with affinity. This result is puzzling because these peptides readily penetrate cells, are proteolytically stable, and co-localize with RNA inside cells. The low cellular activity can be partially explained by the dual mechanism of action of the peptides, which inhibit both initiation of RT and Tat-dependent trans-activation (31). We observed no improvement in RT inhibition biochemically (Figure 3C), suggesting that once TAR RNA is saturated with peptide, maximal RT inhibition is achieved.

However, although JB181 is an extremely potent inhibitor of Tat binding to TAR RNA, it only weakly inhibits loading of the SEC core (P-TEFb, AFF4 and Tat) on TAR *in vitro*. Remarkably, JB181 binds to TAR when the SEC core is also bound, by displacing the Tat-ARM while permitting CycT1 to interact normally with the apical loop of TAR RNA. In other words, inhibition of the Tat ARM by a peptide that binds to the UCU bulge with pM affinity is insufficient to permit strong inhibition of the recruitment of P-TEFb, once it is assembled with Tat. This observation might also explain the weak activity (mid μM) of low nM small molecules synthesized by us in the past (51,56).

Binding of Tat to TAR is important not only because Tat increases the affinity of P-TEFb for TAR is due to its interaction with the CycT1-TRM, but also because it induces a conformational change in TAR that facilitates binding of CycT1 (Figure 6). The direct interaction of its ARM with TAR may simply be required to induce the RNA structural change, while imparting minimal additional affinity. Thus, the dominant contribution to binding energy comes from the recognition of the loop by the CycT1-TRM and the structural rearrangement in the RNA induced by Tat (Figure 6) (8,9,11). Here, we observe binding of an ultra-high affinity mimic of the Tat-ARM to the TAR UCU bulge positions the loop to bind the TRM. Additionally, the Tat-ARM might be functionally more important in the absence of CycT1, to bring Tat (and P-TEFb) to TAR, and in extracting P-TEFb from 7SK RNA where it is stored in an inactive form.

This model for TAR recognition, in which affinity is driven primarily by binding of CycT1 and Tat provides a trigger for RNA conformational changes, is consistent with the long standing observations that mutations in the TAR sequence which disrupt Tat binding have only modest effects on P-TEFb/AFF4/Tat binding to TAR (11). The implication is that targeting the UCU bulge alone, which has been the universally pursued strategy to inhibit TAR, is unlikely to lead to a pharmacological response unless CycT1 interactions with the rearranged TAR RNA loop is also targeted.

## DATA AVAILABILITY

Authors have submitted the atomic coordinates and experimental have been deposited in the Protein Databank along with Biological Magnetic Resonance Bank with accession codes 6D2U and 30452 respectively.

## Supplementary Material

Supplementary Data

## References

[1] Weeks K.M., Ampe C., Schultz S.C., Steitz T.A., Crothers D.M. Fragments of the HIV-1 Tat protein specifically bind TAR RNA. Science. 1990; 249:1281–1285. 2205002 10.1126/science.2205002

[2] Aboul-ela F., Karn J., Varani G. Structure of HIV-1 TAR RNA in the absence of ligands reveals a novel conformation of the trinucleotide bulge. Nucleic Acids Res. 1996; 24:3974–3981. 8918800 10.1093/nar/24.20.3974 PMC146214

[3] Dethoff E.A., Petzold K., Chugh J., Casiano-Negroni A., Al-Hashimi H.M. Visualizing transient low-populated structures of RNA. Nature. 2012; 491:724–728. 23041928 10.1038/nature11498 PMC3590852

[4] Puglisi J.D., Chen L., Frankel A.D., Williamson J.R. Role of RNA structure in arginine recognition of TAR RNA. Proc. Natl. Acad. Sci. U.S.A. 1993; 90:3680–3684. 7682716 10.1073/pnas.90.8.3680 PMC46365

[5] Tao J., Chen L., Frankel A.D. Dissection of the proposed base triple in human immunodeficiency virus TAR RNA indicates the importance of the Hoogsteen interaction. Biochemistry. 1997; 36:3491–3495. 9131998 10.1021/bi962259t

[6] Huthoff H., Girard F., Wijmenga S.S., Berkhout B. Evidence for a base triple in the free HIV-1 TAR RNA. RNA. 2004; 10:412–423. 14970387 10.1261/rna.5161304 PMC1370937

[7] Puglisi J.D., Chen L., Blanchard S., Frankel A.D. Solution structure of a bovine immunodeficiency virus Tat-TAR peptide-RNA complex. Science. 1995; 270:1200–1203. 7502045 10.1126/science.270.5239.1200

[8] Gu J., Babayeva N.D., Suwa Y., Baranovskiy A.G., Price D.H., Tahirov T.H. Crystal structure of HIV-1 Tat complexed with human P-TEFb and AFF4. Cell Cycle. 2014; 13:1788–1797. 24727379 10.4161/cc.28756 PMC4111725

[9] Tahirov T.H., Babayeva N.D., Varzavand K., Cooper J.J., Sedore S.C., Price D.H. Crystal structure of HIV-1 Tat complexed with human P-TEFb. Nature. 2010; 465:747–751. 20535204 10.1038/nature09131 PMC2885016

[10] Madore S.J., Cullen B.R. Genetic analysis of the cofactor requirement for human immunodeficiency virus type 1 Tat function. J. Virol. 1993; 67:3703–3711. 8389901 10.1128/jvi.67.7.3703-3711.1993 PMC237733

[11] Schulze-Gahmen U., Echeverria I., Stjepanovic G., Bai Y., Lu H., Schneidman-Duhovny D., Doudna J.A., Zhou Q., Sali A., Hurley J.H. Insights into HIV-1 proviral transcription from integrative structure and dynamics of the Tat:AFF4:P-TEFb:TAR complex. Elife. 2016; 5:e15910. 27731797 10.7554/eLife.15910 PMC5072841

[12] Abulwerdi F.A., Shortridge M.D., Sztuba-Solinska J., Wilson R., Le Grice S.F., Varani G., Schneekloth J.S. Jr Development of Small Molecules with a Noncanonical Binding Mode to HIV-1 Trans Activation Response (TAR) RNA. J. Med. Chem. 2016; 59:11148–11160. 28002966 10.1021/acs.jmedchem.6b01450 PMC5525537

[13] Davidson A., Begley D.W., Lau C., Varani G. A small-molecule probe induces a conformation in HIV TAR RNA capable of binding drug-like fragments. J. Mol. Biol. 2011; 410:984–996. 21763501 10.1016/j.jmb.2011.03.039 PMC3140652

[14] Davidson A., Leeper T.C., Athanassiou Z., Patora-Komisarska K., Karn J., Robinson J.A., Varani G. Simultaneous recognition of HIV-1 TAR RNA bulge and loop sequences by cyclic peptide mimics of Tat protein. Proc. Natl. Acad. Sci. U.S.A. 2009; 106:11931–11936. 19584251 10.1073/pnas.0900629106 PMC2715490

[15] Hamy F., Felder E.R., Heizmann G., Lazdins J., Aboul-ela F., Varani G., Karn J., Klimkait T. An inhibitor of the Tat/TAR RNA interaction that effectively suppresses HIV-1 replication. Proc Natl Acad Sci U.S.A. 1997; 94:3548–3553. 9108013 10.1073/pnas.94.8.3548 PMC20476

[16] Stelzer A.C., Frank A.T., Kratz J.D., Swanson M.D., Gonzalez-Hernandez M.J., Lee J., Andricioaei I., Markovitz D.M., Al-Hashimi H.M. Discovery of selective bioactive small molecules by targeting an RNA dynamic ensemble. Nat. Chem. Biol. 2011; 7:553–559. 21706033 10.1038/nchembio.596 PMC3319144

[17] Sztuba-Solinska J., Shenoy S.R., Gareiss P., Krumpe L.R., Le Grice S.F., O’Keefe B.R., Schneekloth J.S. Jr Identification of biologically active, HIV TAR RNA-binding small molecules using small molecule microarrays. J. Am. Chem. Soc. 2014; 136:8402–8410. 24820959 10.1021/ja502754f PMC4227816

[18] Eubanks C.S., Forte J.E., Kapral G.J., Hargrove A.E. Small Molecule-Based Pattern Recognition To Classify RNA Structure. J. Am. Chem. Soc. 2017; 139:409–416. 28004925 10.1021/jacs.6b11087 PMC5465965

[19] Patwardhan N.N., Ganser L.R., Kapral G.J., Eubanks C.S., Lee J., Sathyamoorthy B., Al-Hashimi H.M., Hargrove A.E. Amiloride as a new RNA-binding scaffold with activity against HIV-1 TAR. Medchemcomm. 2017; 8:1022–1036. 28798862 10.1039/c6md00729e PMC5546750

[20] Zeiger M., Stark S., Kalden E., Ackermann B., Ferner J., Scheffer U., Shoja-Bazargani F., Erdel V., Schwalbe H., Gobel M.W. Fragment based search for small molecule inhibitors of HIV-1 Tat-TAR. Bioorg. Med. Chem. Lett. 2014; 24:5576–5580. 25466178 10.1016/j.bmcl.2014.11.004

[21] Mei H.Y., Mack D.P., Galan A.A., Halim N.S., Heldsinger A., Loo J.A., Moreland D.W., Sannes-Lowery K.A., Sharmeen L., Truong H.N. Discovery of selective, small-molecule inhibitors of RNA complexes–I. The Tat protein/TAR RNA complexes required for HIV-1 transcription. Bioorg. Med. Chem. 1997; 5:1173–1184. 9222511 10.1016/s0968-0896(97)00064-3

[22] Mei H.Y., Cui M., Heldsinger A., Lemrow S.M., Loo J.A., Sannes-Lowery K.A., Sharmeen L., Czarnik A.W. Inhibitors of protein-RNA complexation that target the RNA: specific recognition of human immunodeficiency virus type 1 TAR RNA by small organic molecules. Biochemistry. 1998; 37:14204–14212. 9760258 10.1021/bi981308u

[23] Wang S., Huber P.W., Cui M., Czarnik A.W., Mei H.Y. Binding of neomycin to the TAR element of HIV-1 RNA induces dissociation of Tat protein by an allosteric mechanism. Biochemistry. 1998; 37:5549–5557. 9548939 10.1021/bi972808a

[24] Richter S., Parolin C., Gatto B., Del Vecchio C., Brocca-Cofano E., Fravolini A., Palu G., Palumbo M. Inhibition of human immunodeficiency virus type 1 tat-trans-activation-responsive region interaction by an antiviral quinolone derivative. Antimicrob. Agents Chemother. 2004; 48:1895–1899. 15105155 10.1128/AAC.48.5.1895-1899.2004 PMC400552

[25] Athanassiou Z., Patora K., Dias R.L., Moehle K., Robinson J.A., Varani G. Structure-guided peptidomimetic design leads to nanomolar beta-hairpin inhibitors of the Tat-TAR interaction of bovine immunodeficiency virus. Biochemistry. 2007; 46:741–751. 17223695 10.1021/bi0619371

[26] Athanassiou Z., Dias R.L., Moehle K., Dobson N., Varani G., Robinson J.A. Structural mimicry of retroviral tat proteins by constrained beta-hairpin peptidomimetics: ligands with high affinity and selectivity for viral TAR RNA regulatory elements. J. Am. Chem. Soc. 2004; 126:6906–6913. 15174860 10.1021/ja0497680

[27] Leeper T.C., Athanassiou Z., Dias R.L., Robinson J.A., Varani G. TAR RNA recognition by a cyclic peptidomimetic of Tat protein. Biochemistry. 2005; 44:12362–12372. 16156649 10.1021/bi0510532

[28] Davidson A., Patora-Komisarska K., Robinson J.A., Varani G. Essential structural requirements for specific recognition of HIV TAR RNA by peptide mimetics of Tat protein. Nucleic Acids Res. 2011; 39:248–256. 20724442 10.1093/nar/gkq713 PMC3017588

[29] Emery F., Bisang C., Favre M., Jiang L.Y., Robinson J.A. A template for the solid-phase synthesis of conformationally restricted protein loop mimetics. Chem. Commun. 1996; 2155–2156.

[30] Favre M., Moehle K., Jiang L.Y., Pfeiffer B., Robinson J.A. Structural mimicry of canonical conformations in antibody hypervariable loops using cyclic peptides containing a heterochiral diproline template. J. Am. Chem. Soc. 1999; 121:2679–2685.

[31] Lalonde M.S., Lobritz M.A., Ratcliff A., Chamanian M., Athanassiou Z., Tyagi M., Wong J., Robinson J.A., Karn J., Varani G. Inhibition of both HIV-1 reverse transcription and gene expression by a cyclic peptide that binds the Tat-transactivating response element (TAR) RNA. PLoS Pathog. 2011; 7:e1002038. 21625572 10.1371/journal.ppat.1002038 PMC3098202

[32] Esteller M. Non-coding RNAs in human disease. Nat. Rev. Genet. 2011; 12:861–874. 22094949 10.1038/nrg3074

[33] Shortridge M.D., Varani G. Structure based approaches for targeting non-coding RNAs with small molecules. Curr. Opin. Struct. Biol. 2015; 30:79–88. 25687935 10.1016/j.sbi.2015.01.008 PMC4416997

[34] Bardaro M.F. Jr, Shajani Z., Patora-Komisarska K., Robinson J.A., Varani G. How binding of small molecule and peptide ligands to HIV-1 TAR alters the RNA motional landscape. Nucleic Acids Res. 2009; 37:1529–1540. 19139066 10.1093/nar/gkn1074 PMC2655691

[35] Bradrick T.D., Marino J.P. Ligand-induced changes in 2-aminopurine fluorescence as a probe for small molecule binding to HIV-1 TAR RNA. RNA. 2004; 10:1459–1468. 15273324 10.1261/rna.7620304 PMC1370632

[36] Brandt S.M., Mariani R., Holland A.U., Hope T.J., Landau N.R. Association of chemokine-mediated block to HIV entry with coreceptor internalization. J. Biol. Chem. 2002; 277:17291–17299. 11782464 10.1074/jbc.M108232200

[37] Levy D.N., Aldrovandi G.M., Kutsch O., Shaw G.M. Dynamics of HIV-1 recombination in its natural target cells. Proc. Natl. Acad. Sci. U.S.A. 2004; 101:4204–4209. 15010526 10.1073/pnas.0306764101 PMC384719

[38] Pearson R., Kim Y.K., Hokello J., Lassen K., Friedman J., Tyagi M., Karn J. Epigenetic silencing of human immunodeficiency virus (HIV) transcription by formation of restrictive chromatin structures at the viral long terminal repeat drives the progressive entry of HIV into latency. J. Virol. 2008; 82:12291–12303. 18829756 10.1128/JVI.01383-08 PMC2593349

[39] Stultz R.D., Cenker J.J., McDonald D. Imaging HIV-1 genomic DNA from entry through productive infection. J. Virol. 2017; 91:e00034-17. 28250118 10.1128/JVI.00034-17 PMC5391475

[40] Hosseinzadeh P., Bhardwaj G., Mulligan V.K., Shortridge M.D., Craven T.W., Pardo-Avila F., Rettie S.A., Kim D.E., Silva D.A., Ibrahim Y.M. Comprehensive computational design of ordered peptide macrocycles. Science. 2017; 358:1461–1466. 29242347 10.1126/science.aap7577 PMC5860875

[41] Gavenonis J., Sheneman B.A., Siegert T.R., Eshelman M.R., Kritzer J.A. Comprehensive analysis of loops at protein-protein interfaces for macrocycle design. Nat. Chem. Biol. 2014; 10:716–722. 25038791 10.1038/nchembio.1580 PMC4138238

[42] Slough D.P., McHugh S.M., Cummings A.E., Dai P., Pentelute B.L., Kritzer J.A., Lin Y.S. Designing Well-Structured cyclic pentapeptides based on Sequence-Structure relationships. J. Phys. Chem. B. 2018; 122:3908–3919. 29589926 10.1021/acs.jpcb.8b01747 PMC6071411

[43] Craik D.J., Fairlie D.P., Liras S., Price D. The future of peptide-based drugs. Chem. Biol. Drug Des. 2013; 81:136–147. 23253135 10.1111/cbdd.12055

[44] Nielsen D.S., Shepherd N.E., Xu W., Lucke A.J., Stoermer M.J., Fairlie D.P. Orally absorbed cyclic peptides. Chem. Rev. 2017; 117:8094–8128. 28541045 10.1021/acs.chemrev.6b00838

[45] Robinson J.A. Beta-hairpin peptidomimetics: design, structures and biological activities. Acc. Chem. Res. 2008; 41:1278–1288. 18412373 10.1021/ar700259k

[46] Srinivas N., Jetter P., Ueberbacher B.J., Werneburg M., Zerbe K., Steinmann J., Van der Meijden B., Bernardini F., Lederer A., Dias R.L. Peptidomimetic antibiotics target outer-membrane biogenesis in Pseudomonas aeruginosa. Science. 2010; 327:1010–1013. 20167788 10.1126/science.1182749

[47] Shortridge M.D., Walker M.J., Pavelitz T., Chen Y., Yang W., Varani G. A macrocyclic peptide ligand binds the oncogenic MicroRNA-21 precursor and suppresses dicer processing. ACS Chem. Biol. 2017; 12:1611–1620. 28437065 10.1021/acschembio.7b00180 PMC5512579

[48] Moehle K., Athanassiou Z., Patora K., Davidson A., Varani G., Robinson J.A. Design of beta-hairpin peptidomimetics that inhibit binding of alpha-helical HIV-1 Rev protein to the rev response element RNA. Angew. Chem. Int. Ed. Engl. 2007; 46:9101–9104. 17893894 10.1002/anie.200702801 PMC3809837

[49] Jadlowsky J.K., Wong J.Y., Graham A.C., Dobrowolski C., Devor R.L., Adams M.D., Fujinaga K., Karn J. Negative elongation factor is required for the maintenance of proviral latency but does not induce promoter-proximal pausing of RNA polymerase II on the HIV long terminal repeat. Mol. Cell. Biol. 2014; 34:1911–1928. 24636995 10.1128/MCB.01013-13 PMC4019061

[50] Belashov I.A., Crawford D.W., Cavender C.E., Dai P., Beardslee P.C., Mathews D.H., Pentelute B.L., McNaughton B.R., Wedekind J.E. Structure of HIV TAR in complex with a Lab-Evolved RRM provides insight into duplex RNA recognition and synthesis of a constrained peptide that impairs transcription. Nucleic Acids Res. 2018; 46:6401–6415. 29961805 10.1093/nar/gky529 PMC6061845

[51] Davis B., Afshar M., Varani G., Murchie A.I., Karn J., Lentzen G., Drysdale M., Bower J., Potter A.J., Starkey I.D. Rational design of inhibitors of HIV-1 TAR RNA through the stabilisation of electrostatic “hot spots”. J. Mol. Biol. 2004; 336:343–356. 14757049 10.1016/j.jmb.2003.12.046

[52] Borkar A.N., Bardaro M.F. Jr, Camilloni C., Aprile F.A., Varani G., Vendruscolo M. Structure of a low-population binding intermediate in protein-RNA recognition. Proc. Natl. Acad. Sci. U.S.A. 2016; 113:7171–7176. 27286828 10.1073/pnas.1521349113 PMC4932932

[53] Hermann T., Westhof E. RNA as a drug target: chemical, modelling and evolutionary tools. Curr. Opin. Biotechnol. 1998; 9:66–73. 9503590 10.1016/s0958-1669(98)80086-4

[54] DeJong E.S., Luy B., Marino J.P. RNA and RNA-protein complexes as targets for therapeutic intervention. Curr. Top. Med. Chem. 2002; 2:289–302. 11944821 10.2174/1568026023394245

[55] Dethoff E.A., Chugh J., Mustoe A.M., Al-Hashimi H.M. Functional complexity and regulation through RNA dynamics. Nature. 2012; 482:322–330. 22337051 10.1038/nature10885 PMC3320162

[56] Murchie A.I., Davis B., Isel C., Afshar M., Drysdale M.J., Bower J., Potter A.J., Starkey I.D., Swarbrick T.M., Mirza S. Structure-based drug design targeting an inactive RNA conformation: exploiting the flexibility of HIV-1 TAR RNA. J. Mol. Biol. 2004; 336:625–638. 15095977 10.1016/j.jmb.2003.12.028

