# Correction to ‘An ultra-high affinity ligand of HIV-1 TAR reveals the RNA structure recognized by P-TEFb’

**DOI:** 10.1093/nar/gkac127

**Published:** 2022-02-21

*Nucleic Acids Research*, Volume 47, Issue 3, 20 February 2019, Pages 1523–1531, https://doi.org/10.1093/nar/gky1197

In the original published version, two peak identification labels in Figure 2A are incorrectly positioned. The labels ‘G36’ and ‘G18’ are reversed. All information regarding these and the remaining peaks in the text remain correct. A new Figure with the correct labels is provided below. This change does not affect the results, discussion and conclusions presented in the article.

**Figure 2.**
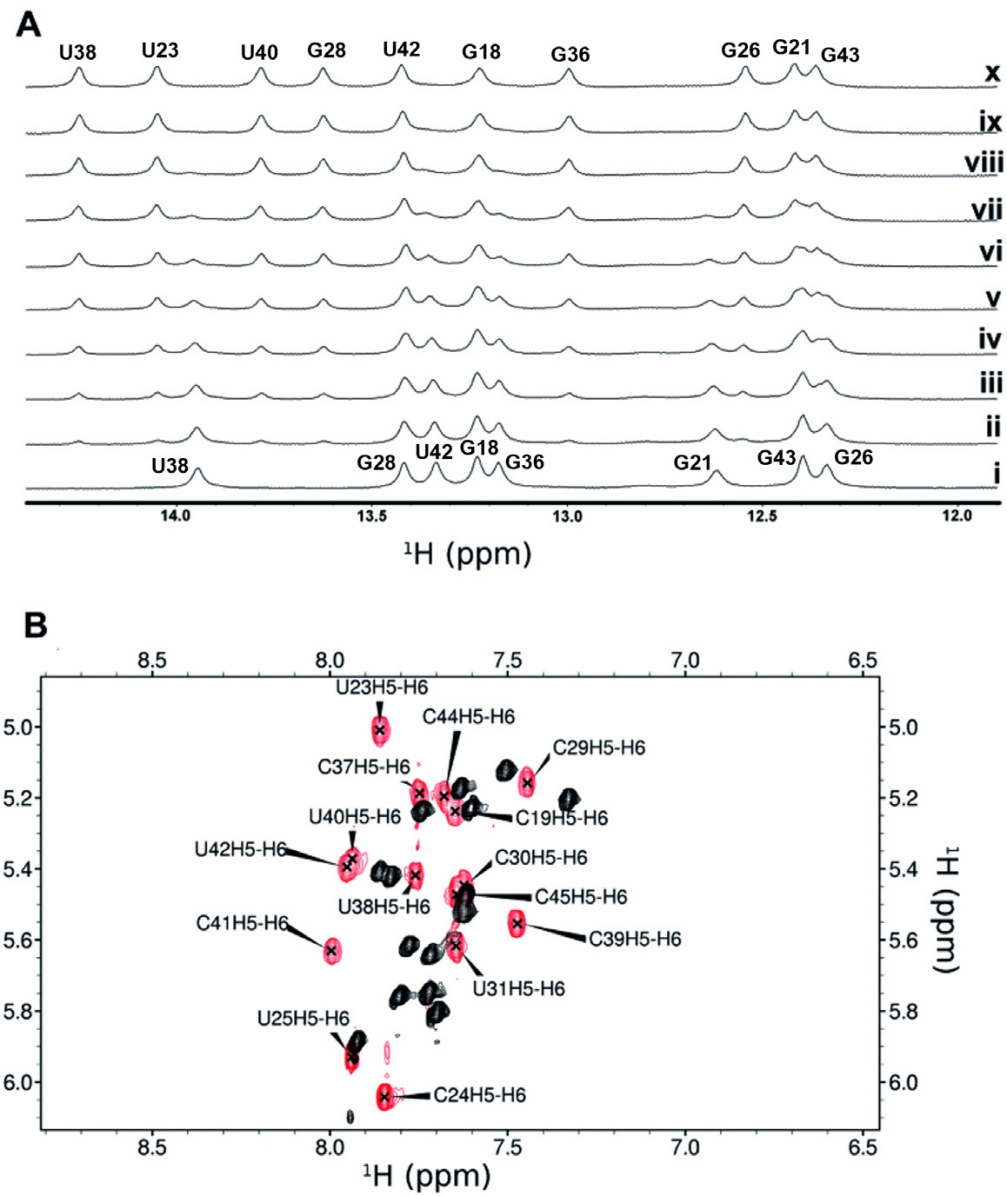
The improvement in binding affinity over other peptides of this class is highlighted by (**A**) clear slow exchange of imino resonance during a titration of HIV TAR with JB181 {1 mM RNA with 0–1.3 mM JB181 (i-x)} and (**B**) large chemical shift changes between free (black) and bound (red) TOCSY spectra. Data were recorded on a 500 MHz Bruker DRX with TCI cryoprobe, at 10◦C in 95%H2O/5%D2O (A) or 25◦C in 99% D2O(B) with the sameNMRbinding buffer conditions (10mMpotassium phosphate pH 6.5, 10 mM sodium chloride, 0.01mM ethylenediaminetetraacetic acid).

