# Supplementary material for: An ultra-high affinity ligand of HIV-1 TAR reveals the RNA structure recognized by P-TEFb
Source: Nucleic Acids Res. 2018 Nov 27;47(3):1523–31. doi: 10.1093/nar/gky1197 (PMC6379670; doi:10.1093/nar/gky1197)
Supplement: Supplementary Data [file gky1197_supplemental_files.pdf]

Supplementary Information for

An ultra-high affinity inhibitor of HIV-1 TAR reveals the RNA structure recognized by P-TEFb

Matthew D. Shortridge*¶, Paul T. Wille*¶¶, Alisha N. Jones*¶, Amy Davidson¶, Jasmina Bogdanovic¶¶¶, Eric Arts¶¶, Jonathan Karn¶¶, John A.Robinson¶¶¶, and Gabriele Varani¶,$

*These authors contributed equally to this work, ¶Department of Chemistry, University of Washington, Seattle, Washington ¶¶Department of Molecular Biology and Microbiology, Case Western Reserve University, Cleveland, Ohio. ¶¶¶Department of Chemistry, University of Zurich, Zurich, Switzerland.

$ Corresponding author: GV

Department of Chemistry, University of Washington, Seattle, Washington

varani@chem.washington.edu

+ 1 206 543 7113

Key words: HIV-1 TAR, Tat, super elongation complex, crystal structure, transcriptional regulation, macrocycles

This PDF file includes:

- Supplementary text
- Figs. S1 to S12
- Tables S1 & S2
- References for SI reference citations

Supplementary Information Text

Experimental Section

NMR Spectroscopy and Spectral Assignments. General methods for resonance assignments of RNA:peptide complexes have been described elsewhere (1-3). Briefly, NMR data were collected on Bruker 500, 600 and 800MHz spectrometers equipped with HCN cryo-probes. Unlabeled and uniformity $^{13}C/^{15}N$ labeled RNA:peptide titration experiments were conducted at 4 °C using 1D ^{1}H excitation sculpting pulse sequence (4) and 2D ^{15}N-^{1}H HSQC. respectively. Dihedral and distance restrains were predicted from H1'-H2' and H1'-H3' peak intensities in 2D ^{1}H-^{1}H total correlation spectroscopy (TOCSY) with TOCSY mixing times of 40ms and 80ms. 2D ^{1}H-^{1}H nuclear Overhauser effect spectroscopy (NOESY) were collected in D_2O and H_2O buffers (20mM potassium phosphate, 10mM sodium chloride, 10μM ethylenediaminetetraacetic acid (EDTA), pH 6.5) at 25°C and 4°C. NOESY spectra with mixing times from 100ms to 350ms were collected for distance restraints with the intensity and volume of the H5/H6 pyrimidine cross peak at 100ms used to calibrate short distances. All NMR data were processed using Bruker Topspin (3.1) or NMRPipe (5) and visualized with Topspin, Sparky (6) or CCPNMR (7).

After the RNA was fully titrated with the JB181 peptide, initial RNA assignments of the complex were identified by comparing TOCSY and NOESY spectra of the TAR:L22 complex; which showed only small differences. Complete RNA assignment of the TAR:JB181 complex was confirmed using uniform $^{13}C/^{15}N$ heteronuclear labeling of TAR RNA and collecting constant time ^{13}C-3D NOESY-HMQC and ^{15}N-edited-HSQC-NOESY experiments at 800 and 500MHz, respectively. Unambiguous hydrogen bonding restraints of RNA base pairs were identified using (Py)H(CC)NN-COSY experiments (8).

Exchangeable and nonexchangeable peptide resonances were initially assigned from the 2D

^{1}H-^{1}H TOCSY and NOESY experiments in H_2O at 800MHz with the majority of peptide backbone HN peaks sufficiently resolved from RNA. Peptide resonances which overlapped with RNA aromatic or sugar resonances were resolved using double filtered. *F1f:F2f* type NOESY experiments collected in H_2O or D_2O NMR buffer. Intermolecular NOEs between the peptide and RNA were clearly observed and resolved in D_2O NMR buffer, the only exception being the HN for Dab1 and Arg8 which overlap aromatic RNA resonances; here strong cross-strand hydrogen bonding allowed for the observation of HN resonances even in 99.99% D_2O buffer.

Structure Determination. The structure of HIV-1 TAR:JB181 complex (pdb entry 5V29) was calculated with XPLOR-NIH (9) with torsion angle dynamics and simulated annealing from an extended starting structure using similar strategy for the TAR peptide complexes (2, 10). The structure was initially folded with NOE-derived distance restraints from 2D ^{1}H-^{1}H NOESY and ^{13}C-edited 3D NOESY-ctHMQC experiments as well as standard hydrogen bonding and base-pair planarity restraints for the positioning of unambiguously established base pairs, to calculate 200 structures. Refinement of this lowest energy structures followed similar approaches described previously using 165 dihedral angle restraints and database potential functions (2, 9-11) but without residual dipolar couplings (RDC). The quality of the NOESY data provided sufficient number of restraints that RDC refinement was not necessary. Convergence was established when we observed no NOE violation greater than 0.5A or dihedral angle violations greater than 5 degrees for the majority of structures. The top twenty converged structures are superimposed in Figure 3b and visualized with PyMol (12).

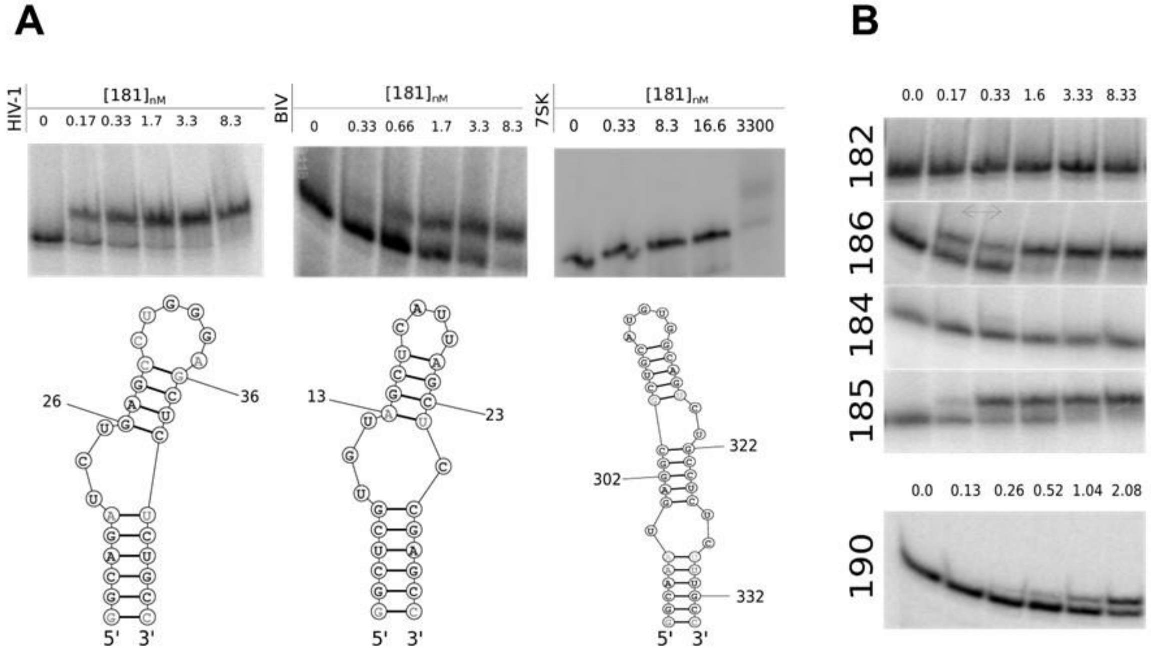

Fig. S1. a) HIV-1 TAR, BIV TAR and 7SK-SL4 RNAs have similar secondary structures, but the JB181 peptide discriminates between them. EMSA reveals that JB181 binds to HIV-1 TAR (<200 pM) much more tightly than BIV TAR (3.3 nM) or 7SK (>3 μM) RNAs. **b**) EMSA of representative JB-peptides with ^{32}P radiolabeled HIV-1 TAR RNA ([RNA]=75pM). The peptide concentration increases from left to right, as indicated. The dissociation constant can be estimated by the peptide concentration where there is an approximate 1:1 ratio (as established by visual inspection) between free RNA and complex, since the RNA concentration is much lower than the K_D.

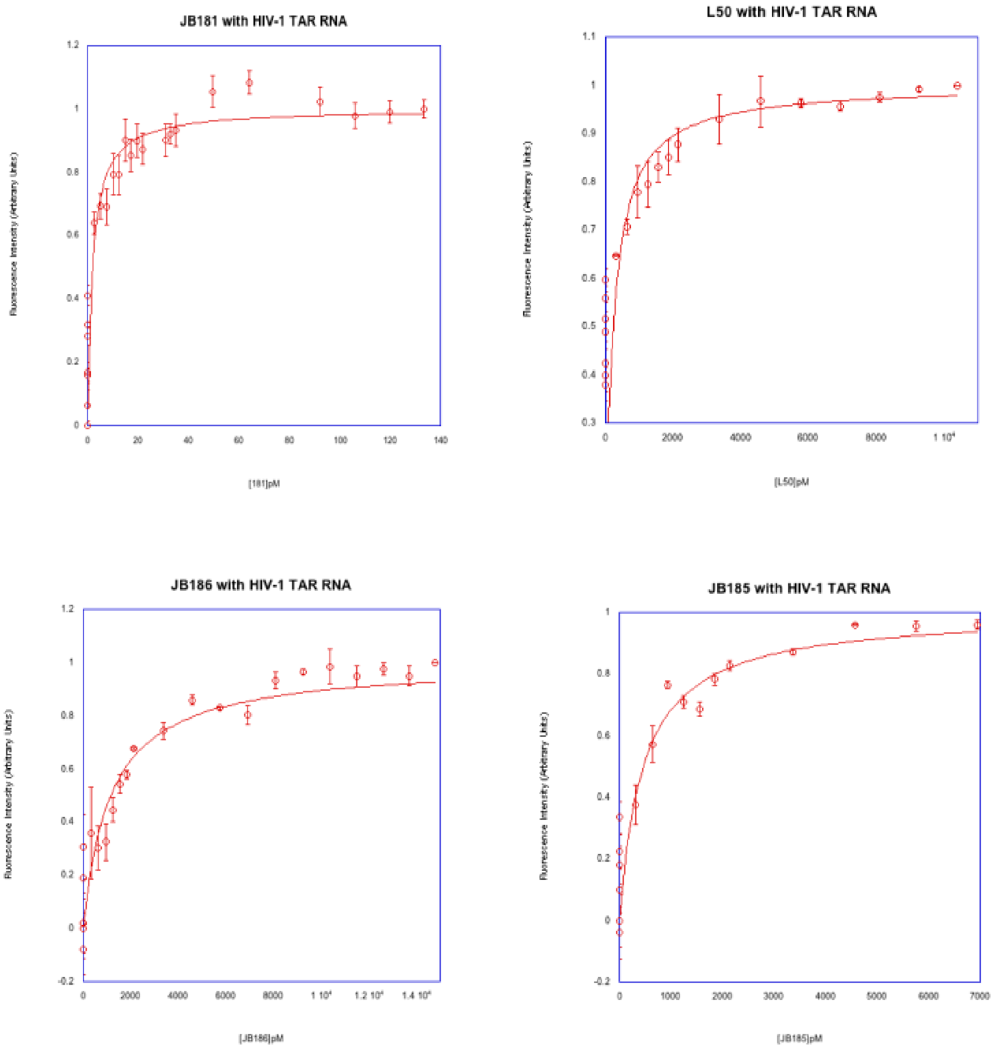

Fig. S2. Binding of JB-peptides to HIV-1 TAR RNA. Normalized fluorescence intensity emitted at 420 nm is plotted vs peptide concentration (pM). The error bars represent the average standard deviation of triplicate titrations. The smooth curve, determined by nonlinear least-squares fitting, shows the best fit through the data points, from which the binding affinity is obtained. Data points whose error bar deviates significantly from the line of best fit probably reflect poor mixing and/or insufficient time for equilibrium to be reached.

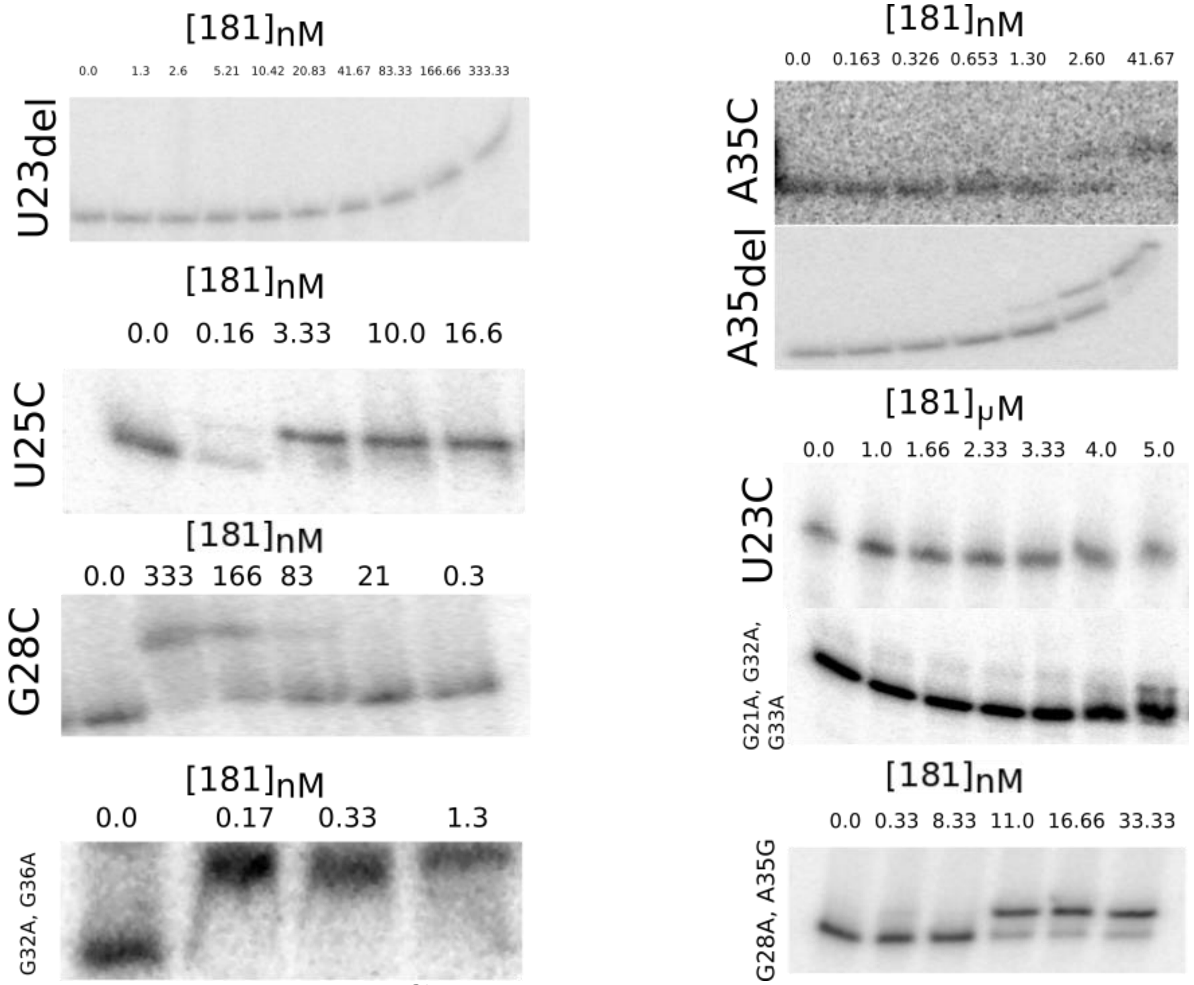

Fig. S3. EMSA of JB181 with ^{32}P radiolabeled HIV-1 TAR RNA variants. The concentration of peptide increases from left to right, as indicated. The dissociation constant can be estimated by the peptide concentration where there is an approximate 1:1 ratio (as established by visual inspection) between free RNA and complex, since the RNA concentration is much lower than the K_d.

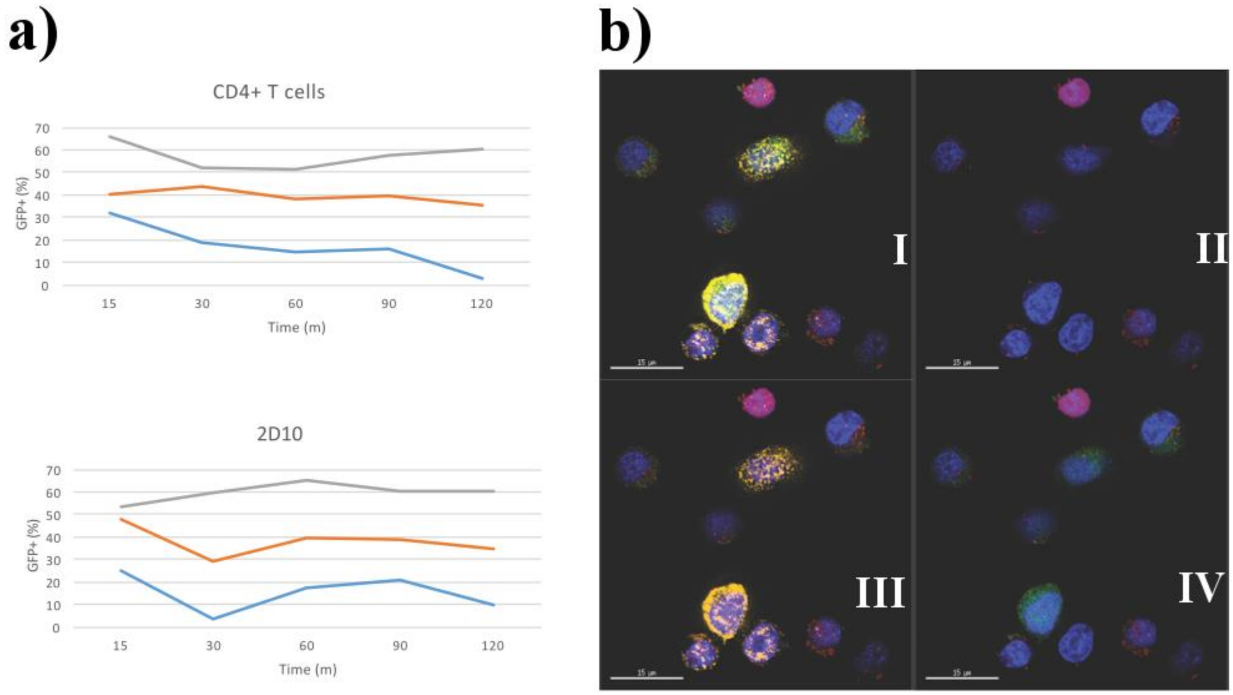

Fig. S4. Uptake of fluorescein-labeled L22. **a).** The turn residue, Lys6, of L22 was first selectively labeled with 5/6-carboxyfluorescein using NHS coupling chemistry and incubated with either CD4+ T cells (top) or 2D10 Jurkat cells (bottom) at 1 µg/mL (blue) 10 µg/mL (orange), or 100 µg/mL (grey). Percent of cells containing peptide was analyzed by flow cytometry. **b).** 2D10 Jurkat cells were first stimulated with TNFα for 4hrs then incubated with L22-AF647 (red) for 30min prior to FISH staining for HIV TAR RNA (yellow), nuclear staining with DAPI (blue) and GFP expression dependent on transcription of HIV (green). Panel I overlay all four stains, Panel II is L22-AF647 only; Panel III is L22-AF647 and TAR FISH, Panel IV L22-AF647 and GFP expression.

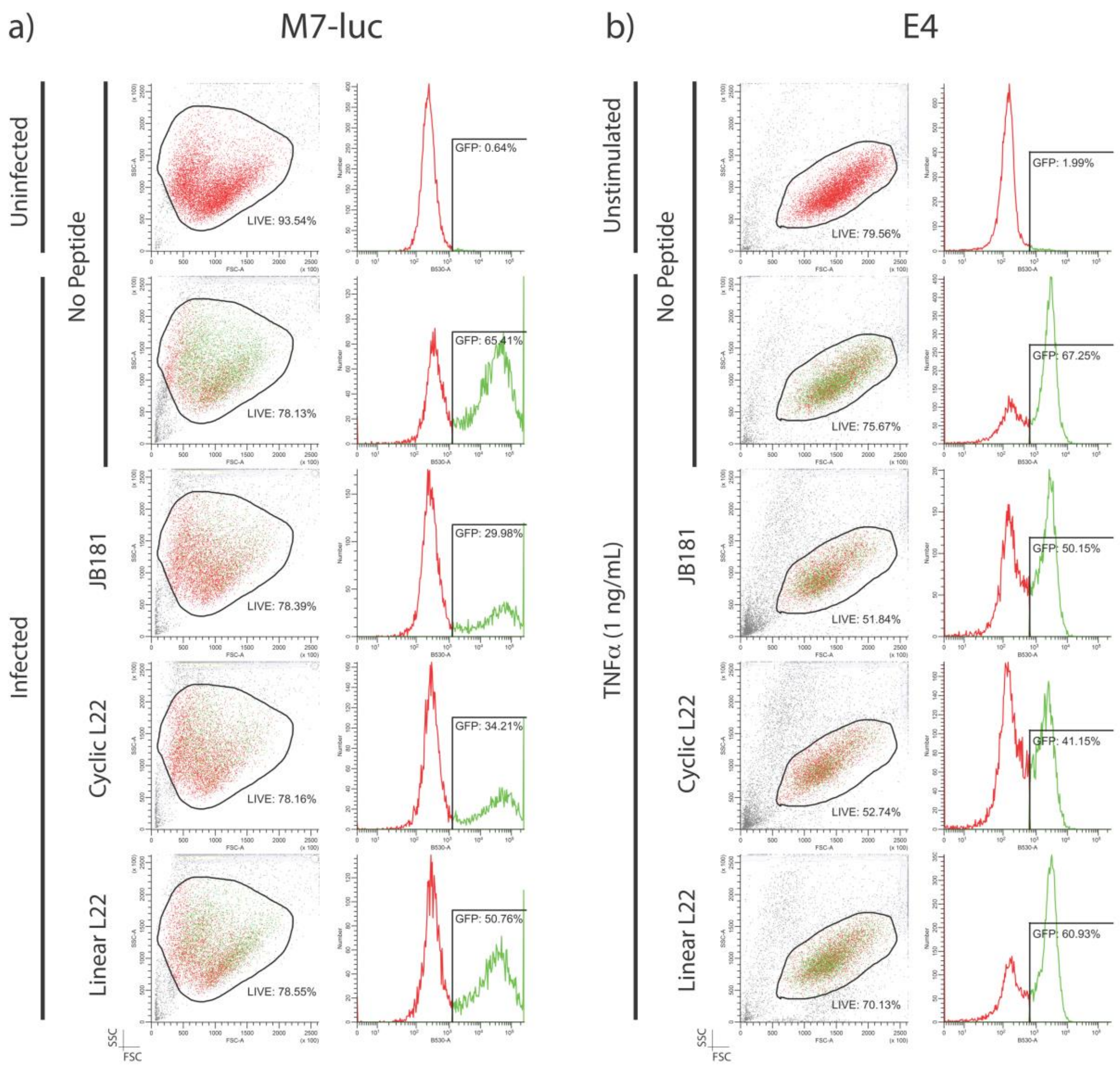

Fig. S5. Representative dot plots and histograms from Figure 7. FSC v. SSC dot plots include live cell gates. Histograms for B-530A channel include gate representing GFP+ cells. a) Uninfected and infected cells without addition of peptide are shown for panel a) from Figure 7. One of three replicates from 100 μM peptide condition are also shown. b) Unstimulated and TNFα stimulated cells in the absence of peptide are shown for panel b) from Figure 7. One of three replicates from 100 μM peptide condition are also shown.

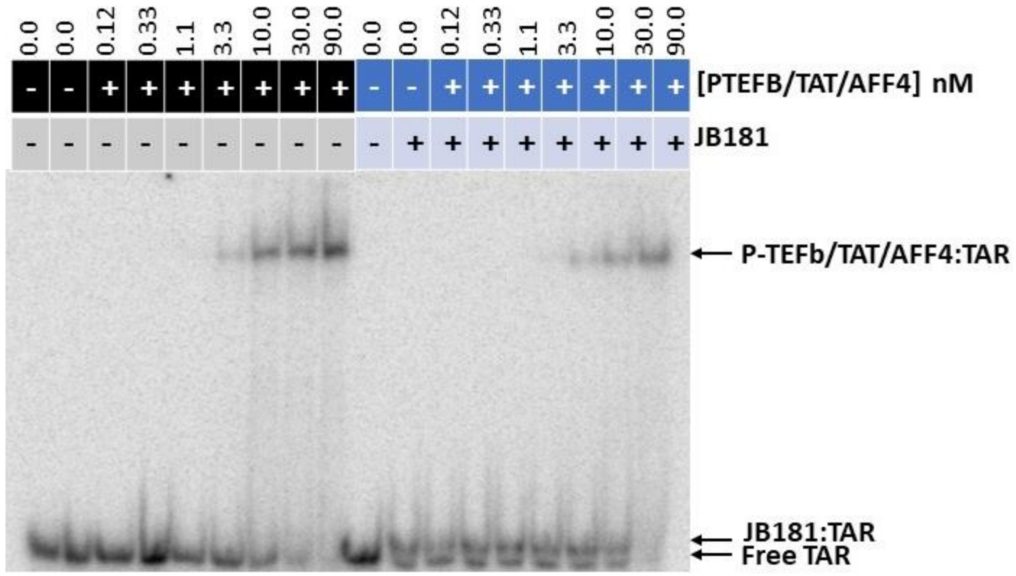

Fig. S6. Representative gel comparing binding affinity of the P-TEFb/TAT1:57/AFF4 to Free HIV-1 TAR (black) or the JB181/TAR preformed complex (blue). Under both conditions, the RNA was held at 100pM and protein complex was increased from 0 to 90nM. The JB181 concentration was held 3-fold excess relative to RNA at 300pM, all sample buffers had 250x fold excess tRNA as background competitor.

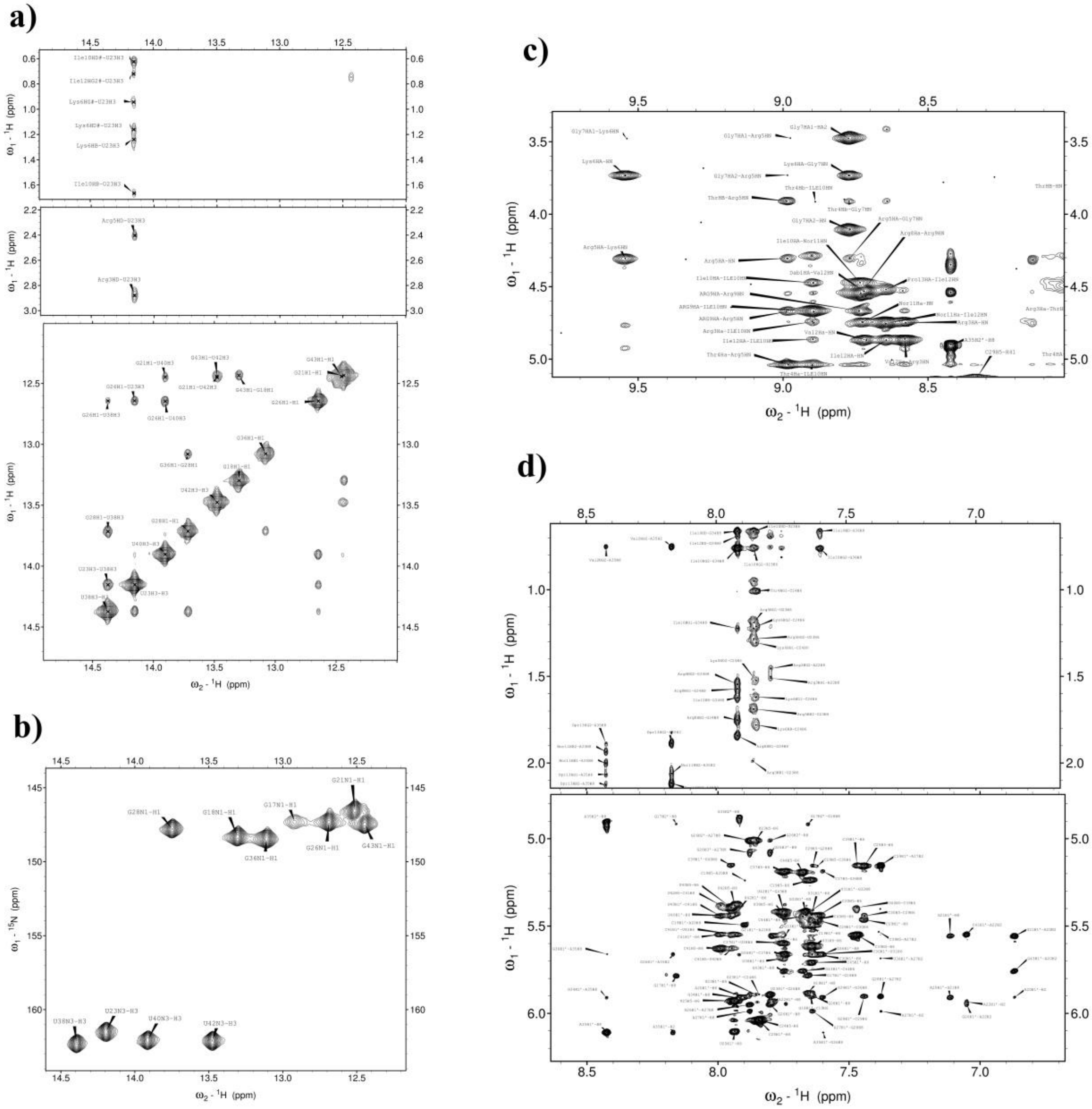

Fig. S7. a) The JB181:HIV-1 TAR complex includes a stable –U23-A27-U38- base triple, as indicated by strong NOEs between U38H3 and U23H3 protons. Furthermore, the imino signal from U23 has eight intermolecular NOEs to JB181 peptide resonances, indicating the peptide directly interacts with the base triple. **b)** The U23 assignment was further confirmed by measuring the ^{15}N chemical shifts. **c)** The peptide remains well folded when bound to TAR, as determined by the Hα chemical shifts and NOE patterns. **d)** The improved binding affinity helped fully resolve many intermolecular NOEs, which allow for proper placement of each side chain relative to the RNA phosphodiester backbone.

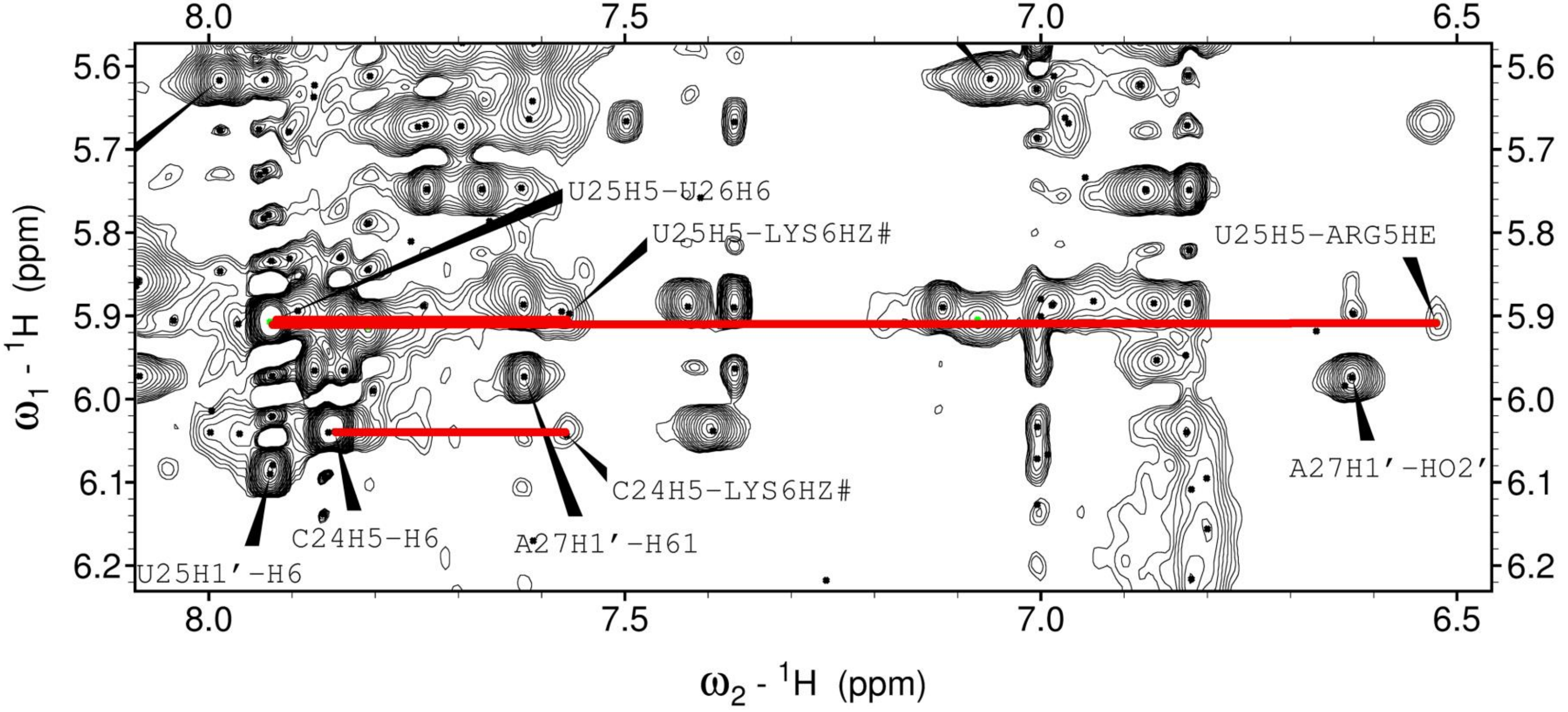

Fig. S8. A close up of the NOESY spectrum for the JB181-TAR complex in H_2O highlining the exchangeable protons involved in proposed hydrogen bond between lysine 6 of JB181 and U25 of HIV TAR. The position of U25 in the JB181 complex is different compared to other TAR structures, this new orientation was established by NOEs from U25-H5 to the Arg5 side chain in both H_2O and D_2O spectra. The greater NOE peak intensity between lysine 6 and U25-H5 compared to C24-H5 suggests the position of the side chain is closer to U25. These structure restraints coupled with mutational analysis of the peptide and RNA suggested we could enforce a hydrogen bond restraint between Lys6 and U25.

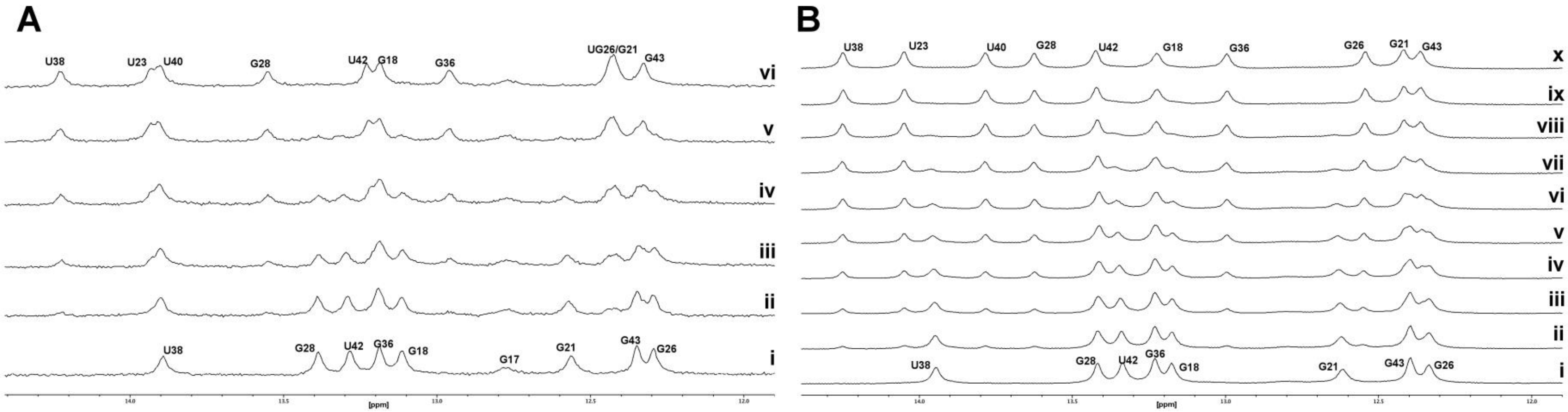

Fig. S9. Cyclic peptide ligands bind very tightly to TAR RNA. With intermediate-slow exchange observed between HIV-1 TAR and L22 (**A**) {150 μM HIV TAR with 0-200 μM L22 (i-vi)} and slow exchange between JB181 and HIV-1 TAR (**B**) {1 mM HIV TAR with 0-1.3 mM JB181 (i-x)}. Both sets of experiments were recorded at 500MHz, at 10 °C in NMR binding buffer (95% H_2O:5% D_2O, 10 mM potassium phosphate pH 6.5, 10 mM sodium chloride, 0.01mM EDTA). The slow exchange observed with the JB181 peptide greatly improved the NMR data quality compared to L22 complex to clearly show the UAU base triple and new contacts between peptide and RNA.

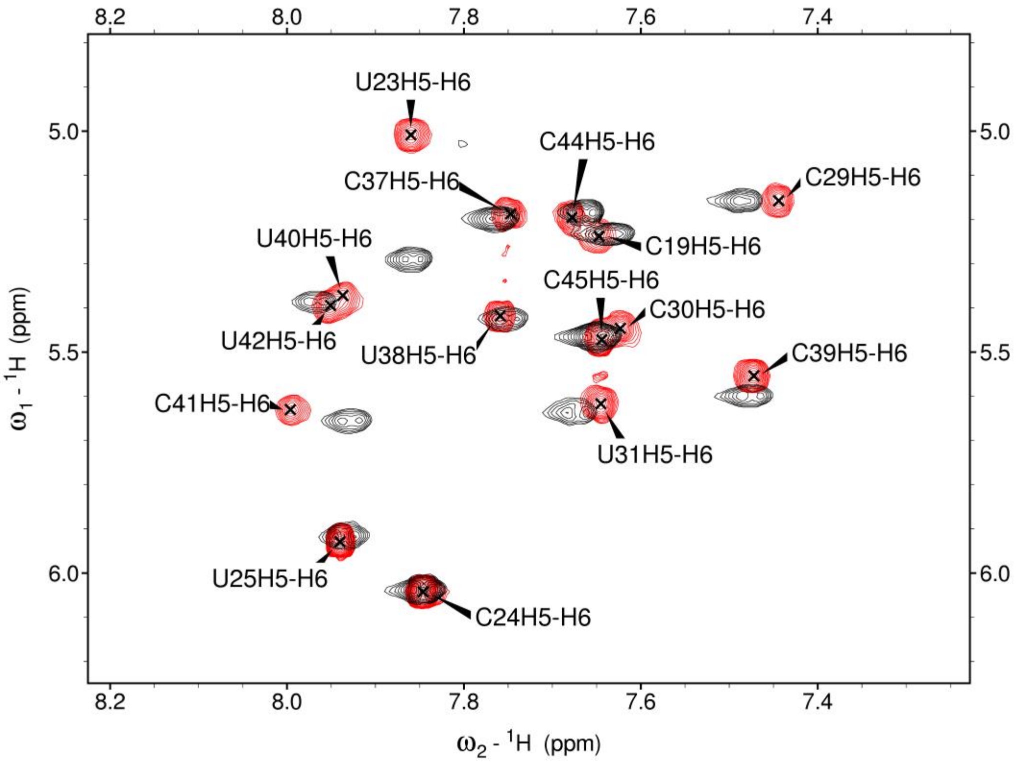

Fig. S10. TOCSY comparison between JB181 (red) and L22 (black). Overall the two datasets are very similar with minor chemical shift difference isolated around the base triple. In the JB181 complex, the U23 peak is clearly visible, where in L22, the peak is barely detectable above noise.

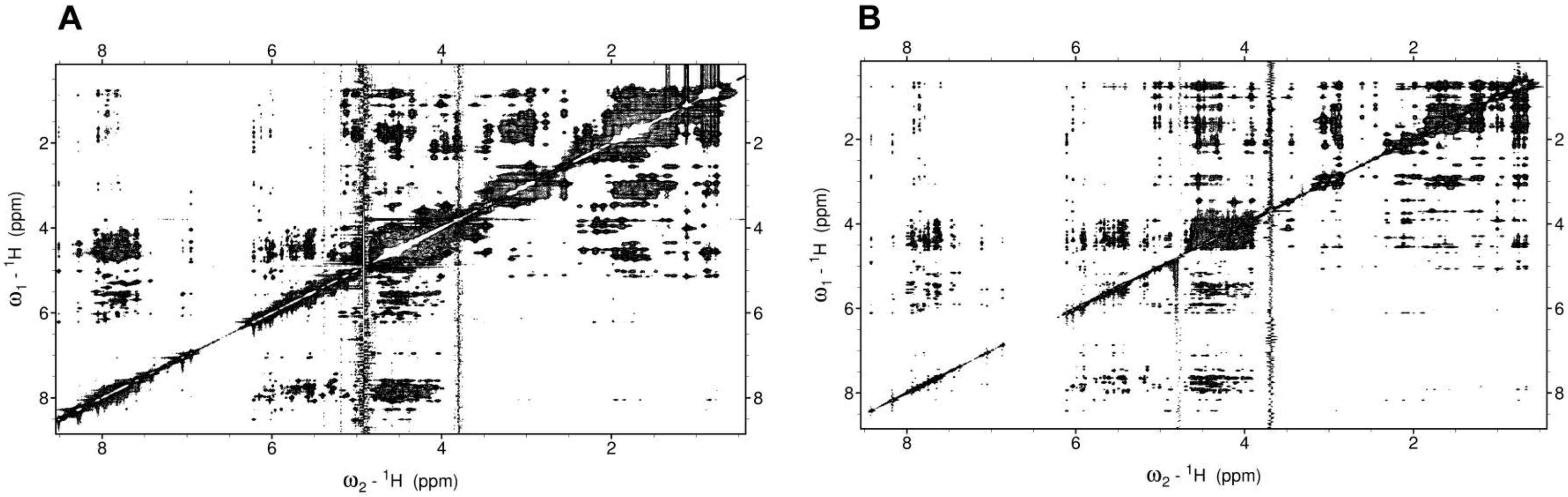

Fig. S11. D_2O NOESY spectra for HIV1-TAR complex with peptide L22 (**A**) or JB181 (**B**) respectively. The two spectra overlap very well with minor differences in chemical shifts for G21-H8, A22-H and A22-H8; these residues are near L-arginine 1 in L22 and L-2-4-diaminobutyric acid in JB181. Many of the intermolecular NOEs are better resolved in spectrum **B** compared to **A** allowing for the precise placement of each side chain.

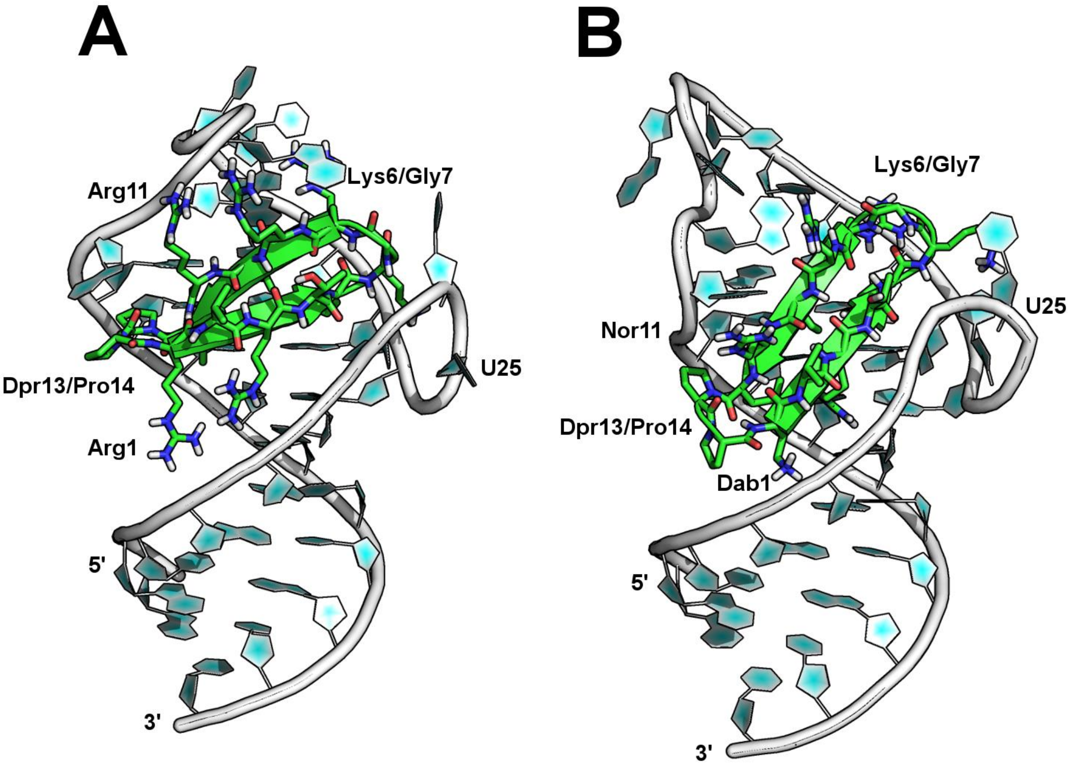

Fig. S12. A side by side comparison between L22 (**A, pdb 5J0M**) and JB181 (**B, pdb 6D2U**) structures shows the peptides are recognized in a similar manner by TAR. However, two specific changes in JB181 led to a very high affinity ligand compared to L22. First, shortening Arg1 sidechain to Dab1 maintained a salt bridge between these cations and RNA backbone. This change shortens the distance between peptide and RNA backbone, pitching the peptide relative to RNA helical axis. Secondly, coupling a short Dab1 with a short Nor11 compared to Arg11 further pitches the peptide, opening a new binding pocket for Arg3 between A22 and U23. These two changes lead to the formation of a new hydrogen bond between Lys6 and U25.

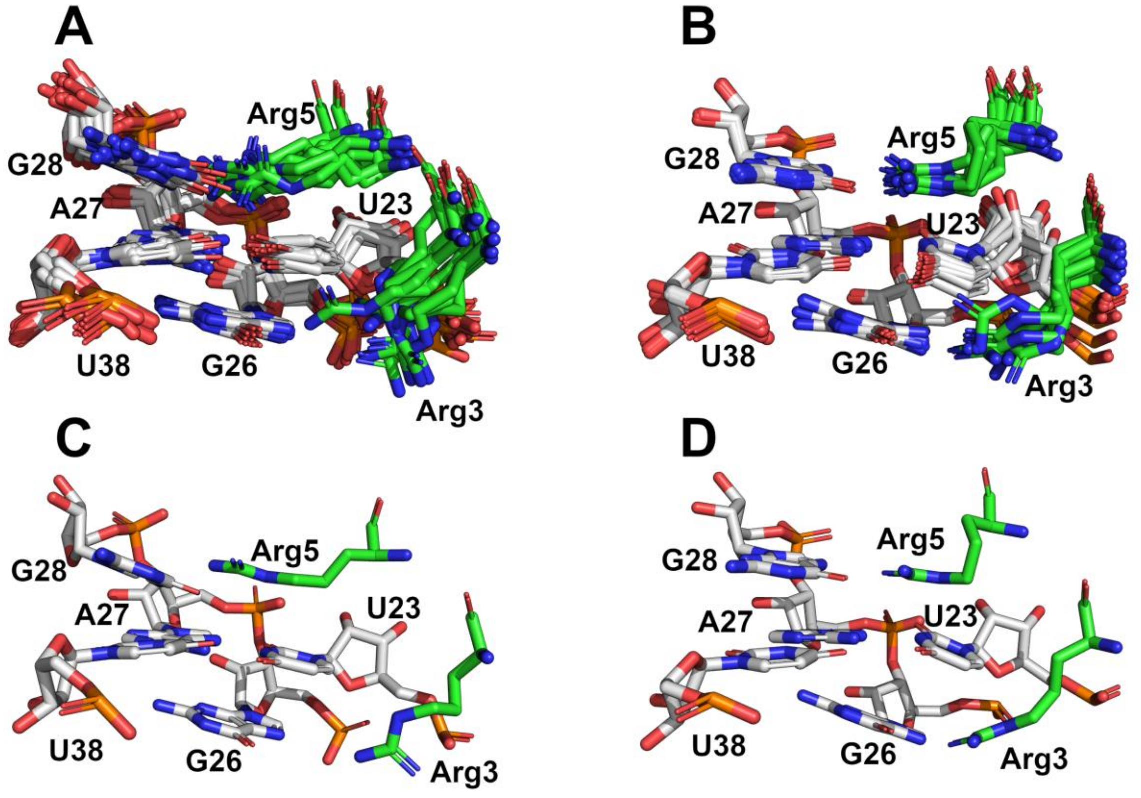

Fig. S13. A close up of the base triple of HIV-TAR with L22 bound (A, C) or JB181 (B,D). Both peptides form hydrogen bond with G28 but JB181 structure is greatly more resolved. In addition to forming base triple with G28 and Arg5, in JB181 Arg3 stacks under U23 to interact with G26. This arginine "sandwiching" helps restrain the base triple as reported in the NMR data (Fig S7). In L22, Arg3 is pointed out towards the RNA backbone, rather than stacked under U23.

Table S1. Binding affinity for a positional scanning library of peptide derivatives of L-22 binding to HIV-1 TAR RNA as determined by EMSA. Position 13 and 14 for each peptide is D-Proline and L-Proline, respectively. Peptides with binding affinities greater than 1 uM were not evaluated further for activity against HIV without tRNA or BIV TAR RNA.

	**Position**												Kd (nM)		
	1	***2***	***3***	***4***	***5***	***6***	***7***	***8***	***9***	***10***	***11***	***12***	w/tRNA	no tRNA	BIV TAR
JB-60	K	V	R	T	R	K	G	R	R	I	R	I	300	3	>5000
JB-61	Q	V	R	T	R	K	G	R	R	I	R	I	>1000		
JB-62	R	Y	R	T	R	K	G	R	R	I	R	I	150	1-5	10
JB-63	R	N	R	T	R	K	G	R	R	I	R	I	100	1	<1
JB-64	R	D	R	T	R	K	G	R	R	I	R	I	>1000		
JB-65	R	T	R	T	R	K	G	R	R	I	R	I	50	1	<1
JB-66	R	A	R	T	R	K	G	R	R	I	R	I	100	1	1
JB-67	R	V	R	T	Q	K	G	R	R	I	R	I	>1000		
JB-68	R	V	R	T	Y	K	G	R	R	I	R	I	>1000		
JB-69	R	V	R	T	R	K	G	Y	R	I	R	I	>1000		
JB-70	R	V	R	T	R	K	G	W	R	I	R	I	>1000		
JB-71	R	V	R	T	R	K	G	R	K	I	R	I	>1000		
JB-72	R	V	^{D}R	T	R	K	G	R	R	I	R	I	>1000		
JB-73	R	V	R	T	R	K	G	R	Y	I	R	I	>1000		
JB-74	R	V	R	T	R	K	G	R	W	I	R	I	>1000		
JB-75	R	V	R	T	R	K	G	R	R	A	R	I	300	5	1-5
JB-76	R	V	R	T	R	K	G	R	R	V	R	I	<50	1	1
JB-77	R	V	R	T	R	K	G	R	R	I	H	I	>1000		
JB-78	R	V	R	T	R	K	G	R	R	I	Y	I	>1000		
JB-79	R	V	R	T	R	K	G	R	R	I	W	I	>1000		
JB-80	R	V	R	T	noR	K	G	R	R	I	R	I	>1000		
JB-81	R	V	R	T	R	K	G	noR	R	I	R	I	1000	25	10-25
JB-82	R	V	R	T	R	K	G	R	R	I	noR	I	75	0.5	1
JB-83	R	V	R	T	R	K	G	R	R	I	R	A	>1000		
JB-84	R	V	R	T	R	K	G	R	R	I	R	V	50	1	1
JB-85	R	V	R	T	R	K	G	R	R	I	R	L	50	5	1
KP-Y-01	hR	V	R	T	R	K	G	R	R	I	R	I	150		
KP-Y-02	R	V	hR	T	R	K	G	R	R	I	R	I	200		
KP-Y-03	R	V	R	T	hR	K	G	R	R	I	R	I	200		
KP-Y-04	R	V	R	T	R	K	G	hR	R	I	R	I	75		
KP-Y-05	R	V	R	T	R	K	G	R	hR	I	R	I	100		
KP-Y-06	R	V	R	T	R	K	G	R	R	I	hR	I	100	5	1

Table S2. NMR and refinement statistics for JB181-TAR complex

	Peptide	Nucleic acid
**Total NMR distance, planarity and dihedral constraints**	1147	
Distance restraints		
Total NOE	297	486
Intraresidue	162	256
Inter-residue		
Sequential ($\|i-j\| = 1$)	59	154
Nonsequential ($\|i-j\| > 1$)	76	76
Hydrogen bonds	12	63
Peptide–nucleic acid intermolecular	92	
Total dihedral-angle restraints	-	167
Total planarity restraints	-	30
**Structure statistics**		
Violations (mean and s.d.)		
Distance constraints (Å)	0.08 ± 0.006	
Dihedral-angle constraints (º)	0.85 ± 0.17	
Max. distance-constraint violation (Å)	0.092	
Max. dihedral-angle violation (º)	1.2	
Deviations from idealized geometry		
Bond lengths (Å)	0.01 ± 0.0005	
Bond angles (º)	0.98 ± 0.02	
Impropers (º)	0.63 ± 0.014	
**Average pairwise r.m.s. deviation (Å)[a]**		
**Peptide**		
Heavy	1.3	
Backbone	0.5	
**RNA**		
All RNA heavy	1.7	
All RNA backbone	1.4	
TAR RNA core (G18–U23, G26–C29, G36–C44, backbone)		
Base triple (U23-A27-U38, backbone)	0.3	
**Complex**		
All complex heavy (C, N, O, P)	1.7	
All complex backbone	1.4	

[a]Pairwise r.m.s. deviation was calculated among 10 refined structures measured by first aligning all structures to the lowest energy model using VMD(13).

Supplementary References:

1. Aboul-ela F, Karn J, & Varani G (1996) Structure of HIV-1 TAR RNA in the absence of ligands reveals a novel conformation of the trinucleotide bulge. *Nucleic Acids Res* 24(20):3974-3981.
2. Davidson A*, et al.* (2009) Simultaneous recognition of HIV-1 TAR RNA bulge and loop sequences by cyclic peptide mimics of Tat protein. *Proceedings of the National Academy of Sciences* 106(29):11931-11936.
3. Hamy F*, et al.* (1997) An inhibitor of the Tat/TAR RNA interaction that effectively suppresses HIV-1 replication. *Proc Natl Acad Sci U S A* 94(8):3548-3553.
4. Stott K, Stonehouse J, Keeler J, Hwang T, & Shaka AJ (1995) Excitation Sculpting in High-Resolution Nuclear Magnetic Resonance Spectroscopy: Application to Selective NOE Experiment. *Journal of the American Chemical Society* 117(14):4199-4200.
5. Delaglio F*, et al.* (1995) Nmrpipe - a Multidimensional Spectral Processing System Based on Unix Pipes. *J Biomol Nmr* 6(3):277-293.
6. Goddard TDK, D.G. (2006) Sparky 3University of California, San Francisco).
7. Vranken WF*, et al.* (2005) The CCPN data model for NMR spectroscopy: development of a software pipeline. *Proteins* 59(4):687-696.
8. Dallmann A*, et al.* (2013) Efficient detection of hydrogen bonds in dynamic regions of RNA by sensitivity-optimized NMR pulse sequences. *Angew Chem Int Ed Engl* 52(40):10487-10490.
9. Schwieters CD, Kuszewski JJ, Tjandra N, & Clore GM (2003) The Xplor-NIH NMR molecular structure determination package. *J Magn Reson* 160(1):65-73.
10. Davidson A, Patora-Komisarska K, Robinson JA, & Varani G (2011) Essential structural requirements for specific recognition of HIV TAR RNA by peptide mimetics of Tat protein. *Nucleic Acids Res* 39(1):248-256.
11. Clore GM & Kuszewski J (2003) Improving the accuracy of NMR structures of RNA by means of conformational database potentials of mean force as assessed by complete dipolar coupling cross-validation. *J Am Chem Soc* 125(6):1518-1525.
12. Schrodinger, LLC (2015) The PyMOL Molecular Graphics System, Version 1.8.
13. Humphrey W, Dalke A, & Schulten K (1996) VMD: visual molecular dynamics. *J Mol Graph* 14(1):33-38, 27-38.
